# Design of a Low-Decomposition–Temperature Polyurethane Foam

**DOI:** 10.1021/acsomega.6c02500

**Published:** 2026-06-15

**Authors:** Nicholas M. Marshall, Gavin DeJong, Connor Murrell, Cade Willis, Mark Kranjc

**Affiliations:** † University of South Carolina Aiken, Department of Chemistry, 471 University Parkway, Aiken, South Carolina 29801, United States; ‡ 1073 Savannah River National Laboratory, Savannah River Site, Aiken, South Carolina 29808, United States

## Abstract

In this work, we describe polyurethane foams designed to degrade at relatively low temperatures, incorporating polypropylene carbonate polyols as well as novel polycarbonates designed for low-temperature degradation. Polyurethane foams formed using degradable polyols and typical water/MDI blowing agents showed poor degradation performance. Significantly better degradation performance was observed in foams formulated to use a minimum of water/isocyanate blowing agent. Degradation performance was also improved by the inclusion of a strong acid catalyst and the use of aliphatic isocyanates in place of aromatic isocyanates. These degradable polyurethane foams may have applications in low-waste packaging or packaging of hazardous materials.

## Graphical abstract

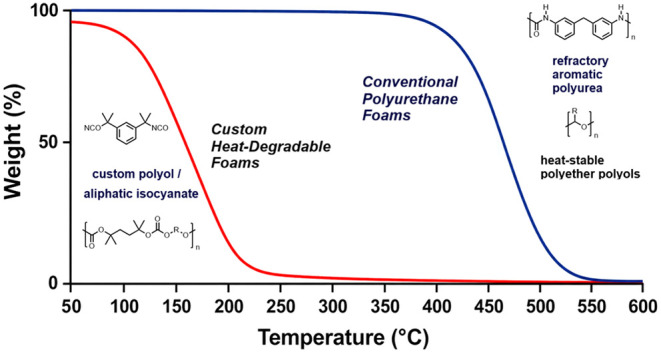

## Introduction

Polyurethanes (PUs), broadly defined as polymers containing repeating R-NH–CO-O-R′ linkages and most commonly formed by the reaction of polyols with polyisocyanate, are a ubiquitous synthetic material used in manufacturing, construction, and packaging.^1–3^ PUs comprise roughly 10% of global plastics manufacturing. While PUs are used for finishes and hardening resins, the single most^4,5^ common application of PU polymers is in the form of foams, prepared by incorporating a blowing agent into the PU formulation.^4^

PUs are thermosetting plastics, second only to polyesters in global usage among thermosets. This property makes PUs immensely useful as padding, insulation, and packaging materials. Uniquely, PUs are often used in a “foam-in-place” (FIP) strategy for packaging irregular or difficult-to-handle objects.^3^ In the FIP approach, the components of a PU foaming formulation are mixed and sprayed into a loaded container, allowing the foam to fill space and secure the load. This strategy would be more broadly applicable if PU foams could be readily decomposed and removed, since a FIP technique could be used to package extremely high-risk cargoes such as radioactive or biohazardous loads. However, like all thermosets, PUs are difficult to decompose and essentially impossible to recycle in actual practice,^5^ although laboratory-scale procedures have been developed including hydrolysis, glycolysis, and aminolysis.^6^

The grand scale of PU manufacturing, along with its intractability toward recycling and slow degradation, creates challenges in the modern manufacturing environment. In keeping with modern principles of green chemistry encouraging “design for degradation,”^7^ and specifically toward the goal of preparing a PU foam that could be used to secure a hazardous load in a package, then readily decomposed to release the load, we sought to design a PU foam which would decompose at an unusually low temperature; ideally, no higher than 110 °C, enabling this practical application in “no-touch” securing and releasing of highly hazardous loads to minimize personnel exposure as required by current regulatory environments.^8^ To our surprise, little published work exists toward the development of a polyurethane which degrades at low temperatures. Extant work in this area is focused around the incorporation of labile polyol linkages (e.g., aliphatic azo moieties). This strategy, while yielding materials with degradation at low onset temperatures, still leave a large char mass residual at high temperatures.^9^ The majority of existing work on PU degradation focuses on photochemical,^9–11^ chemical, or biological degradation or recycling strategies.^6,12,13^ Thus, a need exists for further development of PU foams which fully volatilize at relatively low temperatures. Toward this end, we examined the considerable literature on transient materials, particularly those used for lithographic masking.^14,15^ We found that polycarbonates had been developed that degrade at temperatures as low as 180 °C,^16,17^ and further that the addition of an acid catalyst has been demonstrated to lower the decomposition temperature of certain polycarbonates to a remarkable 65–80 °C. These temperatures are well below the maximum temperature which could be feasibly used to release a secured load, and easily attainable in an industrial polymer recycling or feedstock/fuel conversion process. In this project, we set out to design a polyurethane displaying the properties of low degradation onset temperature as well as low char mass residual, by preparing custom polycarbonate polyols and incorporating them into PU foams with varied isocyanate types and stoichiometries. Following work in transient polycarbonates, we designed or selected from the literature several polycarbonate polyols to incorporate R–OCO bonds in which alkyl R+ carbocations, with or without the presence of acid, are stabilized by resonance or alkyl substitution, reducing the barrier to heterolysis (Scheme 1).

**Scheme 1:**
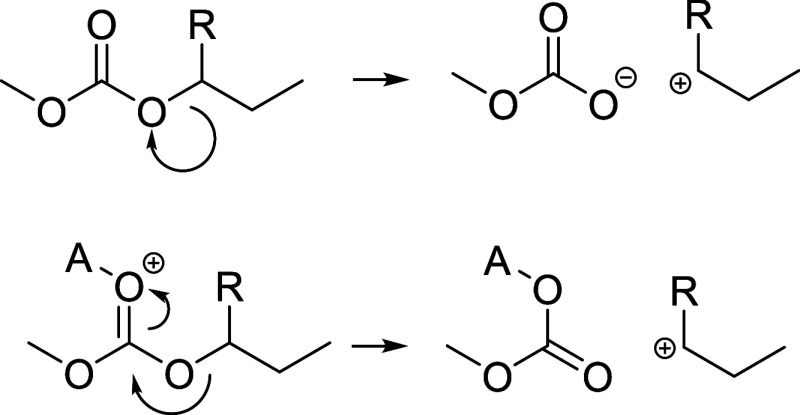
Identity of α-R Groups in Alkyl Polycarbonate Polyols, along with the Effect of an Acid Catalyst A, Affects the Rate of Heterolysis of the Polyol and the Thermal Degradation of Materials Containing It

We also investigated the possibility of preparing polycarbonates with quaternary centers capable of rearranging to tertiary carbocations in the thermal degradation process, especially including the neopentyl glycol (NPG) repeat unit. While these polymers containing “latent” tertiary carbocations displayed improved degradation properties compared to commercial polyols in some cases, we did not find evidence that the proposed rearrangement mechanism was active. Based upon well-established thermal degradation mechanisms of polycarbonates,^18,19^ it is likely that the improvement occurred instead due to the volatility of the diol monomer and that a backbiting mechanism was dominant in the thermal degradation of these materials.

Using the combined mechanism-derived strategy of (a) utilizing polycarbonates and isocyanates capable of degrading to stable carbocations for PU synthesis, (b) minimizing the use of excess isocyanate/water blowing agent, and (c) adding a strong Bronsted-Lowry acid to the formulation, we have successfully produced polyurethane foams with degradation onset temperatures below 90 °C, and which fully volatilize below 250 °C (Scheme 2).

**Scheme 2:**
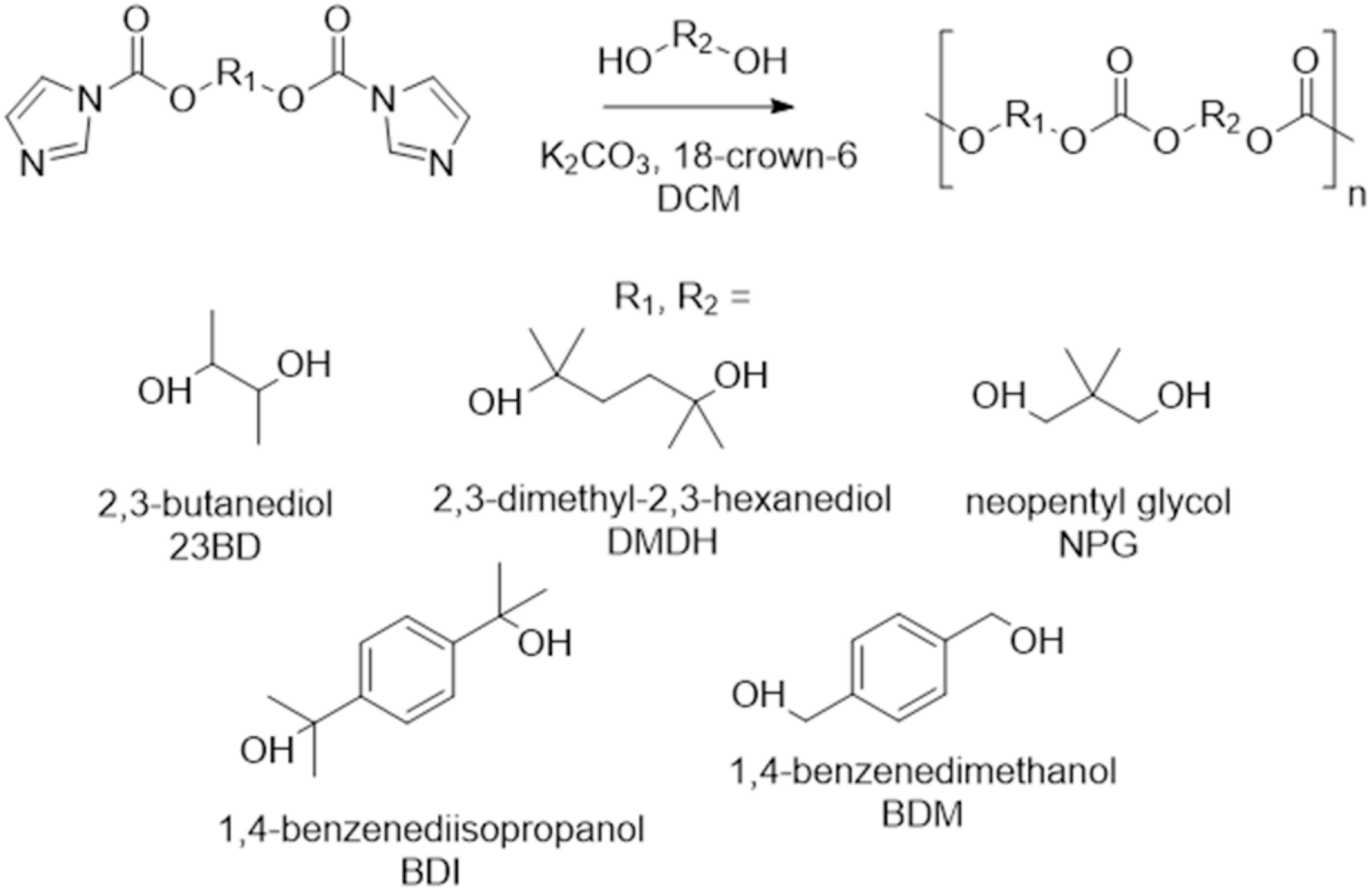
Synthetic Scheme for Polycarbonates Prepared for Use in This Study

## Experimental Section

### General

Unless otherwise stated, all chemicals were obtained from Sigma-Aldrich and used as received. The commercial polyurethane foam used was the pMDI-based FoamIT formulation sold by Smooth-On (Macungie, PA, USA). The azo diol azo­(bis)­isobutanol (AIBDO) was prepared according to a literature method.^16^ Polyurethane formulations were mixed in a fume hood by an operator equipped with a face shield, lab coat, and elbow-length butyl rubber gloves.

### Instrumentation and Simulations

Infrared spectra were acquired on a Nicolet 380 FTIR spectrometer equipped with a DTGS detector and diamond ATR accessory. NMR spectra were acquired using an Anasazi EFT 60 MHz FT-NMR spectrometer referenced to tetramethylsilane at 0 ppm. Thermogravimetric analysis was performed on a Mettler Toledo TGA 2 with a typical temperature range of 20 to 1000 °C at a ramp rate of 20 K/min under air.

### Imidazolate Synthesis

Diimidazolates of diols were prepared based on the method of Coombs et al.^20^ In a typical procedure, 2.93 g (20 mmol, 1.0 equiv) of 2,5-dimethylhexane-2,5-diol was dissolved in 40 mL of sieve-dried dichloromethane, followed by 7.15 g (44 mmol, 2.2 equiv) of carbonyldiimidazole (CDI) and 0.15 g (0.06 equiv) of 4-dimethylaminopyridine (DMAP). The reaction was stirred under nitrogen until complete dissolution of all solids was observed, typically over 12 h. The reaction mixture was transferred to a separatory funnel and extracted three times with deionized water, dried over anhydrous magnesium sulfate, and rotovapped. The resulting fluffy white solid is nearly pure by NMR. The product was recrystallized from a minimum of boiling ethyl acetate and slowly cooled to −20 °C. Vacuum filtration and drying yielded 4.06 g of long white crystals, 61% yield. We found that diimidazolates could be handled in air without noticeable degradation or deliquescence.

#### Titrations

Isocyanates were titrated for total NCO content using the method of W. J. Blank.^21^ OH numbers for commercial and custom polyols were determined using the ASTM D4274–21 standard method,^22^ using acetic anhydride/pyridine as the acylating agent.

#### Products

2,5-dimethylhexane-2,5-diol diimidazolate (61% yield). 1H NMR: (90 MHz, CDCl_3_, δ vs TMS): 1.61 (s,12H), 2.05 (s, 4H), 7.09 (m, 2H), 7.40 (t, 2H), 8.10 (m, 2H) 13C NMR: (23 MHz, CDCl_3_, δ vs TMS): 25, 34, 86, 116, 130, 137, 147.

2,2-dimethyl-1,3-propanediol (neopentyl glycol) imidazolate (97% yield, used without recrystallization); 1H NMR: (90 MHz, CDCl_3_, δ vs TMS): 1.55 (s, 6H), 4.31 (s, 4H), 7.13 (m, 2H), 7.44 (t, 2H), 8.10 (t, 2H). 13C NMR: (23 MHz, CDCl_3_, δ vs TMS): 23, 37, 73, 118, 132, 138, 150.

1,4-benzenediisopropanol imidazolate (58% yield); 1H NMR: (90 MHz, CDCl_3_, δ vs TMS): 1.98 (s,12H), 7.10 (m, 2H) 7.48–7.49 (m,6H: overlapping peaks), 8.12 (t, 2H). 13C NMR: (23 MHz, CDCl_3_, δ vs TMS): 31, 89, 120, 128, 134, 140, 147, 149.

### Polycarbonate Synthesis

Polycarbonates were prepared by the stoichiometric condensation of diols and diimidazolates following the standard procedure of Frechet et al.^23^ In a typical procedure, 6.43 g (19.2 mmol, 1 equiv) of 2,5-dimethylhexane-2,5-diol imidazolate was added to a 100 mL 3-necked flask fitted with a straight water-cooled condenser under nitrogen, followed by 2.66 g (19.2 mmol, 1 equiv) of benzenedimethanol, 16 g (120 mmol, 6.2 equiv) of anhydrous potassium carbonate, and 1 g of 18-crown-6 (3.8 mmol, 0.2 equiv). A total of 30 mL of sieve-dried dichloromethane was added. The heterogeneous mixture was stirred well and heated to gentle reflux. After 12 h, the reaction mixture was filtered, and the solids were washed twice with dichloromethane. For polymers with aromatic components, the combined dichloromethane solution was gravity-filtered into 200 mL of methanol to precipitate the polymer. The collected polymer was washed twice with methanol and vacuum-dried. For wholly aliphatic polymers soluble in methanol, the dichloromethane solution was transferred to a separatory funnel and washed three times with DI water and three times with KCl brine and then dried over anhydrous magnesium sulfate and rotovapped.

#### Products

Poly-1,4-benzenedimethanol-*co*-1,4-benzenediisopropanol carbonate (BDM-*co*-BDI) (31%): Mn 1240 Da, PDI 1.18.

Poly-1,4-benzenedimethanol-*co*-2,5-dimethylhexan-2,5-diol carbonate (BDM-*co*-DMDH) (quant.): Mn 4900, PDI 1.27.

Poly-2,2-dimethyl-1,3-propanediol-*co*-2,5-dimethylhexan-2,5-diol carbonate (DMDH-*co*-NPG) (72%): Mn 410, PDI 2.20.

Poly­(neopentyl glycol carbonate) (NPG homopolymer) (96%): Mn 1300, PDI 1.26.

Poly-2,5-dimethylhexan-2,5-diol-*co*-2,3-butanediol carbonate (DMDH-*co*-23BD) (83%): Mn 1140, PDI 1.57. It contained substantial cyclic polycarbonate as determined by NMR and was not used in foams.

### Polyurethane Foam Formulation

In a typical foam sample preparation, to a 2.5″ × 2.5″ open-topped cube-shaped silicone mold was added 10 g polyol, 0.1 g water, 90 mg dibutyltin dilaurate, 0.2 g silicone surfactant (Gelest DBE-821) and (in applicable cases), plus any other catalysts (pTsOH, DABCO, etc.) or blowing agents. The polyol blend was mixed thoroughly by hand or using a mechanical stirrer with a drill chuck, using a Teflon mixing rod with a spatula tip, until it became a homogeneous slurry (1–2 min). This mixture constituted the formulated polyol. To this mixture was added isocyanate, and mixing was continued for 1 min. The mold was placed in a fume cabinet and allowed to rise and cure overnight.

Example preparation (stoichiometric isocyanate): 10.0 g of Converge 112 PPC, 0.1 g of water, 90 mg of dibutyltin dilaurate, and 0.2 g of DBE-821 were mixed well in a cubic silicone mold, forming a viscous liquid formulated polyol. 3.12 g of tetramethylxylylene diisocyanate (TMXDI) was added, and the liquid blended into the formulated polyol until the mixture was homogeneous. The creamy mixture was allowed to rise to fill the mold and cured overnight at room temperature in a fume hood, forming a rigid white foam, with a density of 1.4 lb/ft^3^, with a closed-cell morphology (Figure S6). Samples for TGA were cut from the interior of the foam cube using a razor blade.

## Results and Discussion

### Observations from TGA

As expected, TGA in air of commercially available foam demonstrated its resistance to direct thermal decomposition (Figure 1). Incorporation of a commercial poly­(propylene carbonate) polyol, Converge 112, in place of the common PEG polyether polyol produced a rigid foam with decomposition properties very similar to the commercial material at low and high temperatures, but which reached 40–50% mass loss at a nearly 100° lower temperature (Figure 1). Encouraged by these initial results, we explored the effects of several additives (1:10 by weight, vs polyol) with the potential to catalyze degradation (Figure S1). While the thermoacids benzeneiodonium tetrafluoroborate and the azo diol AIBDO failed to produce any significant effects on the decomposition of PPC-based foams, the strong Bronsted-Lowry acid *p*-toluenesulfonic acid reduces this foam’s degradation onset temperature by nearly 40°. High-temperature behavior is not significantly altered. We cannot rule out that other radical or thermoacid initiators may be useful degradation catalysts, especially ones with lower initiation temperatures.

**Figure 1:**
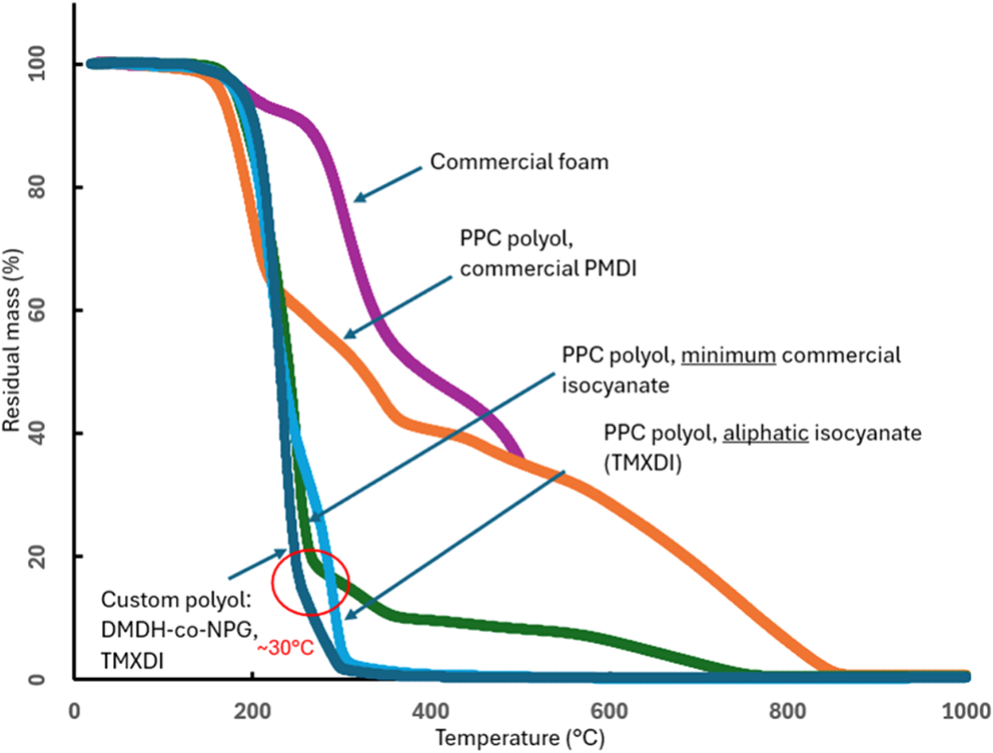
TGA of PU foams showing the effect of changing polyol type and isocyanate fraction on initial and final degradation temperature.

### Interpretation: The Polyurea Hypothesis

Based upon the extensive literature describing thermogravimetric/spectrometric studies of PU foams, and through comparison of PU foam TGA data to TGA of a *bona fide* pure polyurea foam sample (Figure S1) prepared in our laboratory,^24,25^ TGA loss processes of the most common PU foams can be divided into three major regimes. A *volatile product* regime (ca. 80–300 °C) is a nearly sigmoidal loss process in which C–O and C–N bonds begin to undergo heterolysis, and the formation of volatile products begins by elimination. The *polyurea degradation* regime falls, for the common isocyanate MDI, in the range of 230–380 °C (Figure S1). In this range, the urea linkage of the polyurea formed in the reaction of water with two equivalents of isocyanate extrudes a small molecule such as N_2_, CO, or formamide. The result is a cross-linked aromatic material (char) with properties similar to glassy carbon.

Above 380 °C, the early *pyrolysis* stage applies, with formation of hydrocarbon radicals leading to the oxidation and removal of the remaining char. These regimes can be seen clearly in foams produced with commercial two-part mixtures as well as with foams made from polycarbonate polyols using commercial pMDI blends (Figure 1).

In PU foams formulated using the aliphatic isocyanate TMXDI, the polyurea degradation regime becomes a weak shoulder near 250 °C. Based upon other reported studies of aliphatic polyurethane TGA-MS,^26–28^ we believe this change may be due to the introduction of an alternative decomposition pathway by elimination of H_2_NCNHR, through some combination of E1 and E2 processes, to form an alkene breakdown product. In aromatic isocyanates, such elimination is disallowed. This explanation is consistent with the observation of a similar loss process in TGA of a polyol containing a benzenedimethanol (BDM) repeat unit. BDM, lacking a β-hydrogen, cannot undergo elimination and is not lost until reaching a temperature matching its estimated boiling point.^29^ This interpretation is supported by literature reports of BDM-containing polymers (including BDM-*co*-DMDH) releasing BDM during thermal decomposition in TGA-MS.^16^ It is worth noting, furthermore, that the temperature at which the polyurea shoulder appears in this foam is very close to the predicted boiling point of 1,3-diisopropenylbenzene,^29^ the alkene that would be produced by elimination, at 231 °C.On the other hand, the use of the aliphatic isocyanate IPDI (Figure S2 and Scheme 3) produces results slightly inferior (requiring slightly higher temperatures for mass loss) vs TMXDI. While precise elucidation of the reason for this difference would require follow-up TGA-MS studies, it is probable that the primary and secondary C–N bonds in IPDI do not undergo heterolysis as readily as the tertiary benzylic sites in TMXDI. These experimental TGA results are consistent with other literature on TGA of aliphatic PUs^30^ with our molecular design strategy based upon employing isocyanates which readily undergo elimination at the C–N bond, forming relatively volatile alkenesto design low-residue PU foams. Scheme 4.

**Scheme 3:**
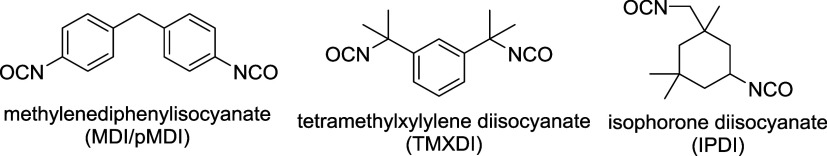
Isocyanates Used in This Study, Comprising Both the Common Commercial Material pMDI and Specialty Production Isocyanates TMXDI and IPDI

**Scheme 4:**
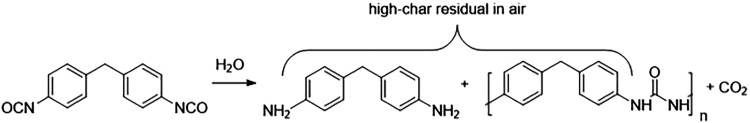
Formation of Aryl Amine and Polyurea Byproducts from Use of Excess Aryl Isocyanate/Water (e.g., pMDI) as a PU Blowing Agent, Yields a Refractory Char Residue in Air

Comparison of the neat and acid-catalyzed polyol TGAs reveals several useful insights (Figures 2–3). Addition of 10% w/w (based on polyol) *p*-toluenesulfonic acid (pTsOH) to the samples produced several significant changes that further elucidate the degradation mechanisms involved in the TGA of these materials. Unsurprisingly, the onset of degradation is lower in all three materials, consistent with literature reports of an E1-type degradation mechanism with a heterolysis step in many polycarbonates.^23^ TGA of commercial PPC in the presence of acid revealed a new process beginning near 120 °C and resulting in a mass loss of ∼20%. This process is linear with time and independent of temperature, consistent with zeroth-order kinetics. The fixed rate of degradation could point to a limited population of catalytically active sites, likely the protonated sites in the polymer, involved in the CO_2_ extrusion process. The total mass lost corresponds to approximately 4.5 mol CO_2_ per mol polymer, implying that nearly half of the carbonate units underwent decarboxylation in this early stage.

**Figure 2:**
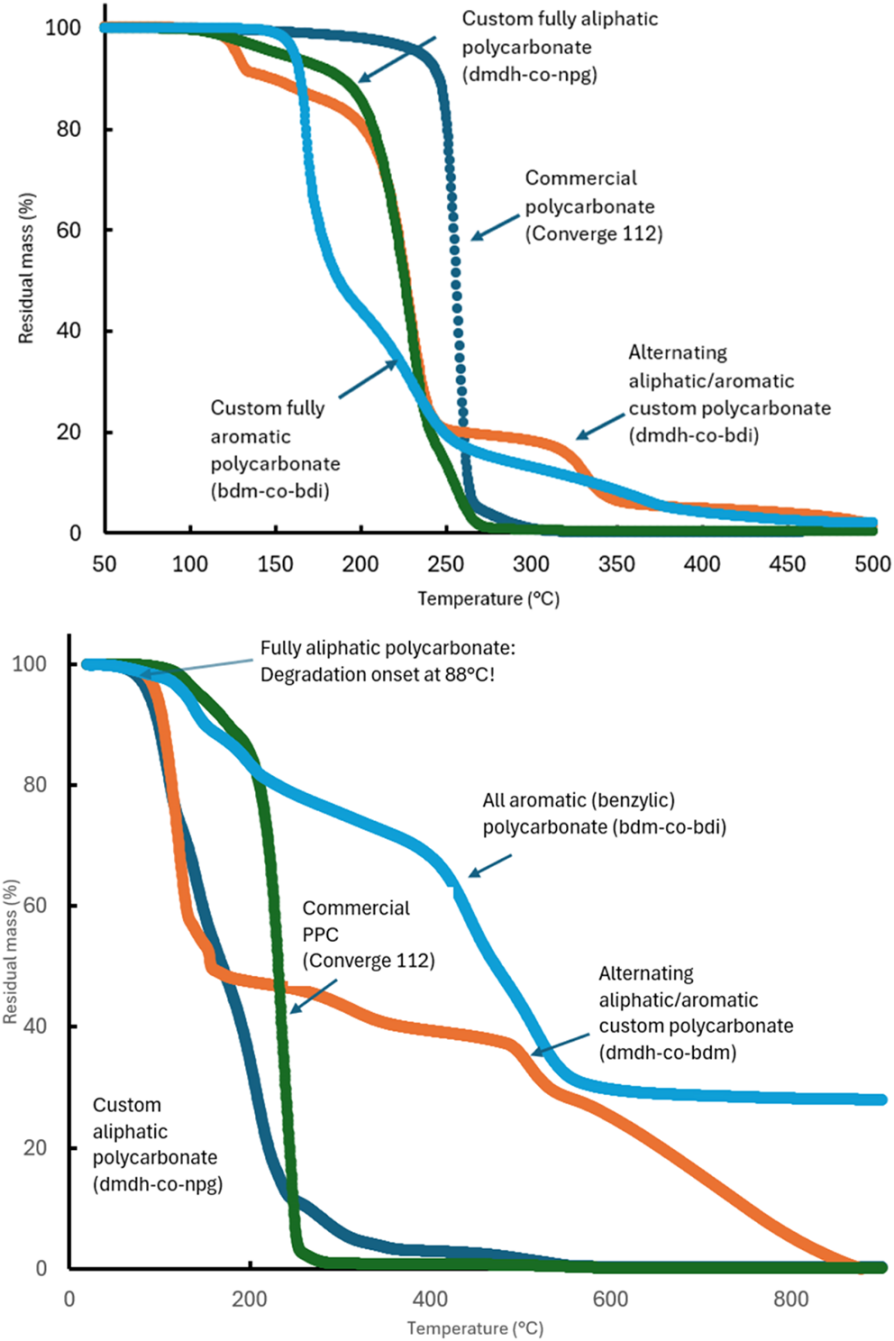
TGA traces of custom polycarbonate polyols with benzylic and tertiary linkages compared to commercial polycarbonate polyols, in the absence (top) and presence (bottom) of 10% pTsOH. The DMDH-*co*-NPG polyol has a higher polydispersity, resulting in a substantial discrepancy between the Mn (ca. 410) and titrated MW (ca. 2000) (Table 1). The resulting foam had a more elastomeric texture and higher cell size variability than foams made with the other polyols synthesized using the same technique.

**Figure 3:**
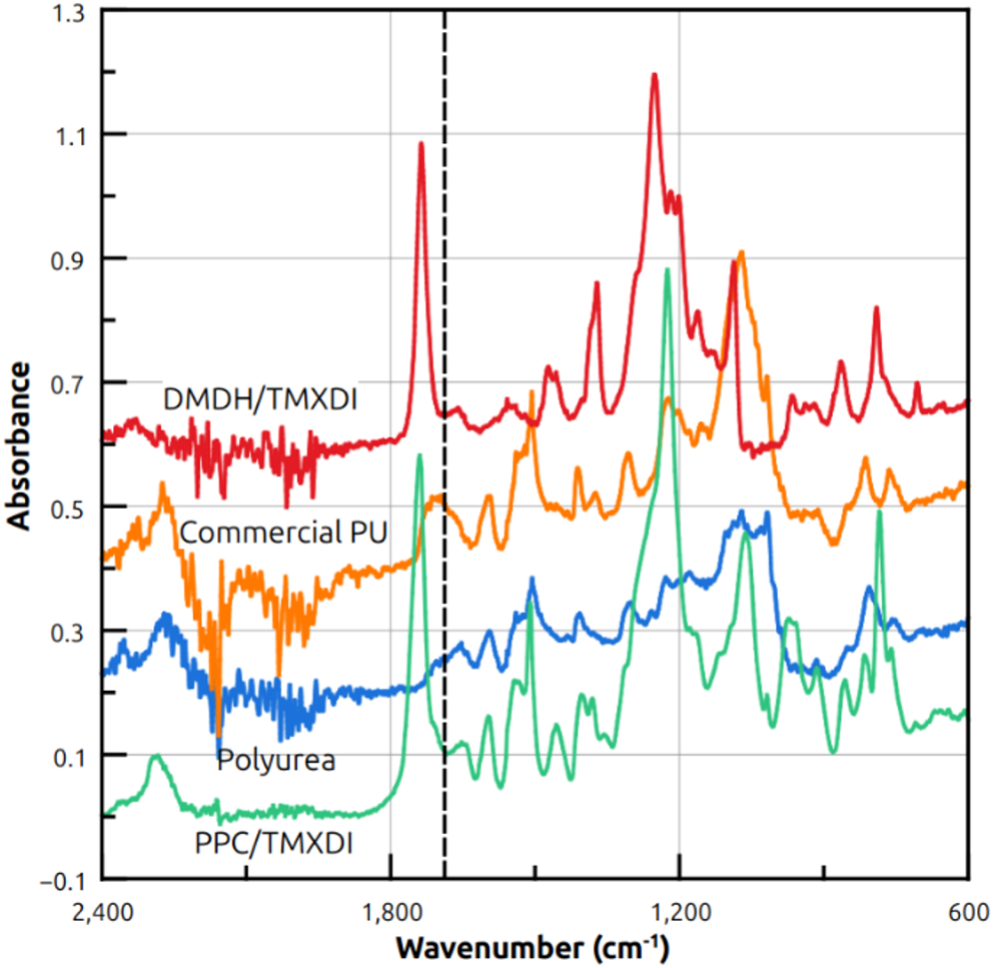
FTIR spectra of commercial and custom PU foams. The polyurea C=O stretching region is indicated by the dashed black line..

As also noted in literature, unmodified PPC decomposes cleanly beginning around 180 °C, making it a potentially useful material as-is for decomposable foams in applications where heating the foam to temperatures achievable by a common household oven is practical. Comparison with the TGA of PU foams formulated with the same PPC polyol (Figure 1) reveals that decomposition of the foam starts some 20 °C lower than the pure polyol, likely due to the effect of residual water and the Lewis acid tin­(II)­laurate. Polyol DMDH-*co*-14BDM initiates decomposition at a lower temperature than PPC, but has a distinct second mass loss regime beginning near 320 °C, with a long residual tail removed only above 500 °C. As previously stated, this phenomenon is likely due to the selective loss of the aliphatic portion of the polymer, leaving the aromatic component to undergo condensation reactions and form a highly unsaturated residue. A theoretical process in which all of the aliphatic components were lost and none of the aromatic compounds was removed would give a residual of 52% based solely on the molar masses of the components (BDM is 158g/mol and DMDH is 146). This value is very close to the first sharp inflection point in the acid-catalyzed degradation of this polymer. Further loss of the OH groups would give a 34% residual, very close to the end of the plateau from 380–490 °C, and finally elimination of this material to pure polyphenylene would yield 23%, which falls somewhere in the first third of the final loss process for this material. Based upon literature studies of other polymers containing benzylic C–O linkages, the refractory^31^ residue observed above 500 °C is a highly aromatic compound similar in structure to a phenolic resin.^32^

Replacement of the benzylic diol BDM with neopentyl glycol (NPG) in the synthesis of the polyol gave the fully aliphatic polyol DMDH-*co*-NPG. We selected NPG as a small diol which can undergo rearrangement processes through methyl 1,2-shifts, ultimately forming isoprene or other rearranged products under dehydrating conditions.^33,34^ This polymer exhibits degradation at a remarkably low onset temperature of 88 °C and continues smooth degradation throughout TGA analysis with less than 1% residual above 270 °C, considerably outperforming PPC and keeping pace with DMDH-*co*-BDM. The as-prepared polymer DMDH-*co*-NPG has a higher polydispersity than the other custom-synthesized polymers in this study, with a substantial shoulder of lower-MW material. While thorough characterization of the mechanical properties of the foams is beyond the scope of this work, we observed qualitatively that DMDH-*co*-NPG-based foams are substantially elastomeric. This unusual property is likely due to the polydisperse nature of this polyol. Based on other work concerning acid-catalyzed aliphatic polycarbonate degradation, we interpret the fully aliphatic DMDH-*co*-NPG TGA with acid to represent three processes (CO_2_ extrusion, loss of eight-carbon diene volatiles from DMDH, and loss of NPG and its elimination products) ending near the boiling point of NPG, 212 °C. Thus, the hoped-for rearrangement-dehydration of NPG to isoprene or other unsaturated volatiles cannot be a major contributor to these decomposition processes under neutral conditions. While this rearrangement-based process cannot be ruled out based solely on the TGA of DMDH-*co*-NPG in the presence of acid, since more mass is lost before 212 °C under acidic conditions, TGA data from NPG homopolycarbonate and its PU foams are inconsistent with NPG dehydration in the decomposition (Figure S3). Remarkably, TsOH *inhibits* thermal decomposition in these materials, shifting the TGA curve of both polyol and foam 20 °C higher with minimal change of the curve shape. Since any decomposition process involving heterolysis of the C–O bond would be catalyzed by acid, the decomposition of NPG-based materials must occur by a different route. A backbiting mechanism, as widely described in polycarbonate literature, is a possible alternative degradation pathway; such a mechanism might be inhibited by the protonation of nucleophilic end group sites, since bases are known to catalyze backbiting.^17,35^

### Titration Results: Establishment of NCO Ratios in the Commercial Material

The titration of commercial isocyanates, commercial polyols, and polyols synthesized in our laboratory allowed us to establish the NCO ratio of each material. Titration results show that the commercial foam studied has a large excess of NCO (Table 1), resulting in rapid foaming and solidification both when used with the commercial polyether polyol and with commercial PPC and copolymer blends in the present study. Variation of the polyol while keeping the NCO index unchanged led to only minor effects on the TGA of the foam products.

**Table 1:** Equivalent Weights of Various Polyols as Measured by Titration^a^

material	MW (g/equiv)	OH number
Foam-IT Pt. B	605	93
Converge 56	2000	56
Converge 112	1000	112
BDM-*co*-DMDH	491	114
DMDH-*co*-NPG	2073	27
NPG homopoly.	471	119
Foam-IT Pt. A (isocyanate)	131	

a Weights are given in g/mol of functional group (NCO in the case of Foam-IT pt. A, OH in other cases). Other, non-polymeric isocyanates were assumed to have the correct MW for their structure. FoamIT Pt. B is a polyether polyol, and Pt. A is a pMDI blend. The theoretical MW/equiv for pure MDI is 125 g/mol NCO.

### Infrared Spectroscopy: Observations of Polyurea in Foams

Comparison of the FTIR spectra of commercial PU foams and foams formulated with similar isocyanate:polyol ratios is consistent with the formation of large quantities of refractory polyurea in the commercial material. Similarly, a control experiment in which the polyol is omitted from a PU foam formulation yields a friable yellow foam, which is presumably polyurea with residual catalyst and surfactant. The polyurea C=O yields several distinct peaks in IR, is distinct from carbonate and polyurethane C=O, allowing the various polycarbonyls to be distinguished qualitatively by IR. Based upon our attribution of refractory char formation in pMDI-based PU foams to high polyurea fraction, we would predict less pronounced PU peaks in materials that lack that TGA feature. This prediction is borne out by data (Figure 3). The broader polyurea C=O peaks at 1700 (C=O) and 1100 (C–N)­are visible but small in the spectrum of the foam designed for minimal isocyanate blowing agent use, consistent with our design hypothesis for these materials.

### Influence of the Methylene Chloride Blowing Agent

Perhaps the most obvious conclusion that can be drawn from these data is that the most important determining factor in the thermal degradation of PU foams is the amount and type of isocyanate used, especially the fraction reacted with water as a blowing agent. It is well-known that isocyanate/water blowing agent systems generate polyurea and free amine byproducts, which are more resistant to thermal degradation than polyurethanes of similar structure due to the greater strength of the C–N bond.^25^ Polyureas from aryl isocyanates such as pMDI and TDI are particularly resistant to thermal decomposition due to the great strength of the aryl ROCN-Ar bond.^25,36^ The TGA of a pure polyurea prepared by direct formulation of pMDI with water demonstrates the thermally refractory nature of this material. (Figure S1). Therefore, strategies for minimizing the need for isocyanate/water blowing agent are needed. The technology of auxiliary blowing agents is well-established in the PU foam literature. In our hands, the incorporation of 30% v/v CH_2_Cl_2_, relative to the volume of commercial PPC polyol, produced a rigid foam approximately 40% larger in volume than the same formulation without additional water/isocyanate blowing agent. However, comparison of TGA results from these materials shows moderate differences in their thermal degradation properties (Figure S4). The onset of degradation in the foam made with CH_2_Cl_2_ is much lower, but some char formation is visible near 300°. These observations are consistent with either the loss of volatile, entrapped CH_2_Cl_2_ with heating of the foam or the degradation of CH_2_Cl_2_ to catalytic HCl under these conditions.^37^ The use of methylene chloride or other volatile organic blowing agents to enable the use of lower NCO/H_2_O fractions and thus minimize the formation of polyureas in PU foams shows some promise.

### Foam Applications and Physical Properties

In a recent report,^38^ we demonstrated based on a finite-element analysis that for the intended purpose of heavy equipment load securement, foam densities of 3–5 lb/ft^3^ are required. By contrast, light shipping materials (e.g., packing peanuts made from foamed polystyrene or starch) have a substantially lower density of 0.2–0.8 lb/ft^3^. Therefore, the foams as prepared in this study (1.4 ± 0.4 lb/ft^3^) are not suitable for common packaging materials. However, the fractions of water, excess isocyanate, and auxiliary blowing agent, such as CH_2_Cl_2_, should be easily variable, allowing for optimization of density through a design-of-experiments approach.

## Conclusions

While common off-the-shelf polyurethane two-part foams do not display substantial degradation over the time frame studied until heated to over 500 °C, PU foams incorporating the polycarbonate polyol PPC degrade at a significantly lower temperature than similar commercial formulations. Including a strong Bronsted-Lowry acid catalyst induces charring and darkening at lower temperatures, but it does not undergo bulk mass loss or structural degradation significantly faster. High heat resistance in common PU foams, desirable for many applications, is due primarily to the presence of the strong aromatic polyurea linkages between MDI subunits. These linkages are the dominant polymeric moiety in most two-part foams due to the large fraction of isocyanate/water blowing agent blend in these formulas.

The incorporation of aromatic compounds in *any* role in PU foam formulations promotes the formation of thermally intractable residues, which may consist of a highly cross-linked unsaturated material similar in structure to phenolic resins. Formation of these residues can be sharply reduced by minimizing the use of water/isocyanate blowing agent in PU foam formulations, but this strategy may not always be useful because of the need to achieve particular mechanical properties of the foam. Residue formation can be largely avoided by molecular design strategies, selecting polyol and isocyanate monomers such that the most labile bonds (C–O and C–N) possess at least one *α*-hydrogen. We ascribe the success of this strategy to E1-style elimination processes in the degrading material, producing relatively volatile alkene or cyclic carbonate byproducts.^39^ Broadly speaking, polyurethane foams can be successfully designed for thermal degradation by ensuring that individual monomer units of the polyol and isocyanate components are low in molecular weight, which may be due to the availability of low-barrier pathways through rearrangement and/or E1 elimination to the formation of volatile compounds.

Based upon these strategies, we report the design of polycarbonate polyols that undergo thermal degradation at lower temperatures than common commercial polyols, particularly the alternating polycarbonate based on neopentyl glycol and 2,5-dimethylhexane-2,5-diol, as well as the polycarbonate homopolymer of neopentyl glycol. For commercial-scale production, optimization using greener and less costly methods of polycarbonate synthesis^40^ will be needed. Combining these polyols with the aliphatic isocyanate TMXDI, capable of E1 elimination to 1,3-bis­(1-methylethenyl)­benzene, we also report PU foams which undergo effective thermal degradation within the range of 110–150 °C. These easily degraded materials and other PU materials designed with a similar strategy may be useful for the packaging of hazardous waste, plastic waste reduction, or to enable the chemical recycling of polyurethanes. Future work in this area will focus on detailed characterization and optimization of the degradable PU foam mechanical and thermal properties (e.g, density, modulus, thermal conductivity) and development of cost-efficient, scalable syntheses of the polycarbonate components.

## Supplementary Material



## Notes

Additional TGA data and original ^1^H/^13^C NMR spectra of polymers and precursors (PDF)

## Abbreviations

PU: polyurethane
FIP: foam-in-place
TGA: thermogravimetric analysis
FTIR: Fourier transform infrared spectroscopy
p-TsOH: para-toluenesulfonic acid
AIBDO: azo-bis­(isobutanediol)
DMAP: 4-dimethylaminopyridine
PPC: polypropylene carbonate
DMDH: 2,5-dimethylhexane-2,5-diol
NPG: neopentyl glycol
IPDI: isophorone diisocyanate
TMXDI: tetramethylxylylene diisocyanate
MDI: meta-diphenylmethanediisocyanate
pMDI: poly-MDI. “*X*-*co*-*Y*” refers to an alternating polycarbonate copolymer of monomers *X* and *Y*. Case is not significant in polymer abbreviations

## References

[1] Kaur R., Singh P., Tanwar S., Varshney G., Yadav S. (2022). Assessment
of Bio-Based Polyurethanes: Perspective on Applications and Bio-Degradation. Macromolecules.

[2] de Souza, F. M. ; Kahol, P. K. ; Gupta, R. K. Introduction to Polyurethane Chemistry. In Polyurethane Chemistry: Renewable Polyols and Isocyanates; ACS Symposium Series; American Chemical Society, 2021; Vol. 1380, pp 1–24 10.1021/bk-2021-1380.ch001.

[3] Szycher’s Handbook of Polyurethanes, 2nd ed.; Szycher, M. , Ed.; CRC Press: Boca Raton, 2012 10.1201/b12343.

[4] Furtwengler P., Perrin R., Redl A., Avérous L. (2017). Synthesis
and Characterization of Polyurethane Foams Derived of Fully Renewable
Polyester Polyols from Sorbitol. Eur. Polym.
J..

[5] Jin F.-L., Zhao M., Park M., Park S.-J. (2019). Recent Trends of
Foaming in Polymer Processing: A Review. Polymers.

[6] Wieczorek K., Bukowski P., Stawiński K., Ryłko I. (2024). Recycling
of Polyurethane Foams via Glycolysis: A Review. Materials.

[7] Anastas P., Eghbali N. (2010). Green Chemistry: Principles and Practice. Chem. Soc. Rev..

[8] NRC: 10 CFR 71.43 General standards for all packages. https://www.nrc.gov/reading-rm/doc-collections/cfr/part071/part071-0043.html. (accessed January 07, 2020).

[9] Hendry, D. G. ; Hill, M. E. ; Peters, H. M. Solid Polymers Thermally Degradable to Flowable Compositions. US Patent US3909497A, 1975 https://patents.google.com/patent/US3909497A/en. (accessed June 21, 2021).

[10] Duden, Q. E. Catalyzed Decomposing Structural Payload Foam. WO Patent WO2006127021A2, 2006 https://patents.google.com/patent/WO2006127021A2/en. (accessed June 21, 2021).

[11] Markle, R. A. ; Elhard, J. D. ; Bigg, D. M. ; Sowell, S. ; Brusky, P. L. ; Cremeans, G. E. Thermally-Reversible Isocyanate-Based Polymers. US Patent US5387667A, 1995 https://patents.google.com/patent/US5387667A/en. (accessed June 21, 2021).

[12] Kemona A., Piotrowska M. (2020). Polyurethane Recycling and Disposal: Methods and Prospects. Polymers.

[13] Liu Y., Zhou H., Guo J.-Z., Ren W.-M., Lu X.-B. (2017). Completely
Recyclable Monomers and Polycarbonate: Approach to Sustainable Polymers. Angew. Chem., Int. Ed..

[14] Camera K. L., Wenning B., Lal A., Ober C. K. (2016). Transient Materials
from Thermally-Sensitive Polycarbonates and Polycarbonate Nanocomposites. Polymer.

[15] Acar H., Çınar S., Thunga M., Kessler M. R., Hashemi N., Montazami R. (2014). Study of Physically Transient Insulating
Materials
as a Potential Platform for Transient Electronics and Bioelectronics. Adv. Funct. Mater..

[16] Gärtner R., Nuyken O., Voit B., Vermeersch J., Van Damme M. (1998). Labile Polycarbonates Containing
Azo Units Susceptible
to Thermolytic or Acidolytic Degradation. Des.
Monomers Polym..

[17] Darensbourg D. J. (2018). Comments
on the Depolymerization of Polycarbonates Derived from Epoxides and
Carbon Dioxide: A Mini Review. Polym. Degrad.
Stab..

[18] Fréchet J. M. J., Bouchard F., Eichler E., Houlihan F. M., Iizawa T., Kryczka B., Willson C. G. (1987). Thermally Depolymerizable Polycarbonates
V. Acid Catalyzed Thermolysis of Allylic and Benzylic Polycarbonates:
A New Route to Resist Imaging. Polym. J..

[19] Resendiz-Lara D. A., Habets T., Armes S. P., Williams C. K. (2026). Controlled Polymerization
Catalysis for the Synthesis of Degradable Amphiphilic Polycarbonates
from CO2. J. Am. Chem. Soc..

[20] Coombs T. C., Lee, Wong H., Armstrong M., Cheng B., Chen W., Moretto A. F., Liebeskind L. S. (2008). Practical,
Scalable, High-Throughput Approaches to
H3-Pyranyl and H3-Pyridinyl Organometallic Enantiomeric Scaffolds
Using the Achmatowicz Reaction. J. Org. Chem..

[21] Blank, W. J. Polymers, Industrial Coatings, Crosslinking. nco_titrat; https://www.wernerblank.com/polyur/testmethods/ncotitrat.htm (accessed 2025–09–25).

[22] ASTM International . Standard Test Methods for Testing Polyurethane Raw Materials: Determination of Hydroxyl Numbers of Polyols. 2021 10.1520/D4274-21.

[23] Houlihan F. M., Bouchard F., Frechet J. M. J., Willson C. G. (1986). Thermally Depolymerizable
Polycarbonates. 2. Synthesis of Novel Linear Tertiary Copolycarbonates
by Phase-Transfer Catalysis. Macromolecules.

[24] Chattopadhyay D. K., Webster D. C. (2009). Thermal Stability and Flame Retardancy of Polyurethanes. Prog. Polym. Sci..

[25] Awad W. H., Wilkie C. A. (2010). Investigation of
the Thermal Degradation of Polyurea:
The Effect of Ammonium Polyphosphate and Expandable Graphite. Polymer.

[26] Yang W. P., Macosko C. W., Wellinghoff S. T. (1986). Thermal
Degradation of Urethanes
Based on 4,4′-Diphenylmethane Diisocyanate and 1,4-Butanediol
(MDI/BDO). Polymer.

[27] Cervantes-Uc J. M., Espinosa J. I. M., Cauich-Rodríguez J. V., Ávila-Ortega A., Vázquez-Torres H., Marcos-Fernández A., San Román J. (2009). TGA/FTIR Studies of Segmented Aliphatic Polyurethanes
and Their Nanocomposites Prepared with Commercial Montmorillonites. Polym. Degrad. Stab..

[28] Grassie N., Zulfiqar M. (1978). Thermal Degradation
of the Polyurethane from 1,4-Butanediol
and Methylene Bis­(4-Phenyl Isocyanate). J. Polym.
Sci. Polym. Chem. Ed..

[29] SciFinder Scholar . Calculated Using ACD/Labs Software. https://scifinder.cas.orgwww.acdlabs.com. (accessed June 28, 2025).

[30] Camadanli S., Hisir A., Dural S. (2022). Synthesis and Performance of Moisture
Curable Solvent Free Silane Terminated Polyurethanes for Coating and
Sealant Applications. J. Appl. Polym. Sci..

[31] Bilow, N. ; Miller, L. J. Aromatic Polymers and Method. US Patent US3578611A, 1971 https://patents.google.com/patent/US3578611A/en. (accessed April 09, 2026).

[32] Jiang H., Wang J., Wu S., Yuan Z., Hu Z., Wu R., Liu Q. (2012). The Pyrolysis Mechanism of Phenol Formaldehyde Resin. Polym. Degrad. Stab..

[33] Perry, M. A. ; De, B. R. E. Preparation of Aldehydes from Diols by a Dehydration Rearrangement Reaction. US Patent US2870214A, 1959 https://patents.google.com/patent/US2870214/en. (accessed April 16, 2026).

[34] Yvernault T., Mazet M. (1967). Acid-Catalyzed Dehydration of 2,2-Dimethylpropane-1,3-Diol and Its
2,2-Disubstituted Derivatives. I. Acid-Catalyzed Dehydration of 2,2-Dimethylpropane-1,3-Diol. Bull. Soc. Chim. Fr..

[35] Nieboer V., Fanjul-Mosteirín N., Olsén P., Odelius K. (2024). Mastering Macromolecular Architecture
by Controlling
Backbiting Kinetics during Anionic Ring-Opening Polymerization. Macromolecules.

[36] Mouren A., Avérous L. (2023). Sustainable
Cycloaliphatic Polyurethanes: From Synthesis
to Applications. Chem. Soc. Rev..

[37] Olek M., Baron J., Zukowski W. (2013). Thermal Decomposition
of Selected
Chlorinated Hydrocarbons during Gas Combustion in Fluidized Bed. Chem. Cent. J..

[38] Kranjc, M. D. ; Murrell, C. ; McKeel, C. A. ; Osborne, M. L. ; Marshall, N. Synthesizing, Compounding, and Characterizing a Heat Labile Foam (LDRD-2024–00016); SRNL-STI-2024–00446; SRS 2024 https://www.osti.gov/biblio/2458207. (accessed April 15, 2026).

[39] Kilian L., Wang Z.-H., Long T. E. (2003). Synthesis and Cleavage of Core-Labile
Poly­(Alkyl Methacrylate) Star Polymers. J. Polym.
Sci. Part Polym. Chem..

[40] Olsson J. V., Hult D., García-Gallego S., Malkoch M. (2017). Fluoride-Promoted
Carbonylation Polymerization: A Facile Step-Growth Technique to Polycarbonates. Chem. Sci..

