# Supplementary material for: Design of a Low-Decomposition–Temperature Polyurethane Foam
Source: ACS Omega. 2026 Jun 15;11(25):37660–7. doi: 10.1021/acsomega.6c02500 (PMC13325135; doi:10.1021/acsomega.6c02500)

Supporting information for

Design of a Low-Decomposition-Temperature Polyurethane Foam

Nicholas M. Marshall[1],* Connor Murrell[2], Cade Willis[2], and Mark Kranjc[2]

1. University of South Carolina Aiken, Department of Chemistry, 471 University Parkway, Aiken, SC.

2. Savannah River National Laboratory, Savannah River Site, Aiken, SC 29808

Foam	Polyol	Isocyanate	Notes	Degrad. onset (°C)	50% degrad. (°C)	Mass residual (%)
Commercial PU	Foam-IT polyester	Foam-IT PMDI	NCO index = 3.3	195	390	35
Polyurea only	water only	Foam-IT PMDI		290	644	35
PPC + commercial PMDI		Foam-IT PMDI		191	295	0.4
PPC + minimum PMDI		Foam-IT PMDI	NCO index = 1	181	241	0.4
PPC + TMXDI	Converge 112	TMXDI		165	235	0.1
PPC + TMXDI + acid	Converge 112	TMXDI	10% TsOH	176	241	0.9
PPC + isophorone	Converge 112	IPDI		183	239	0.3
PPC + isophorone + acid	Converge 112	IPDI	10% TsOH	141	249	1.2
DMDH + TMXDI				139	231	0.2
DMDH + TMXDI + acid			10% TsOH	88	222	1.1
homo-NPG + TMXDI				149	274	0
homo-NPG + TMXDI + acid			10% TsOH	144	291	1.4

Table S1. Degradation onset temperatures, 50% mass loss degradation temperatures, and final mass residuals for foams in Fig. 1, and foams prepared from polyols in Figs. 2-3.

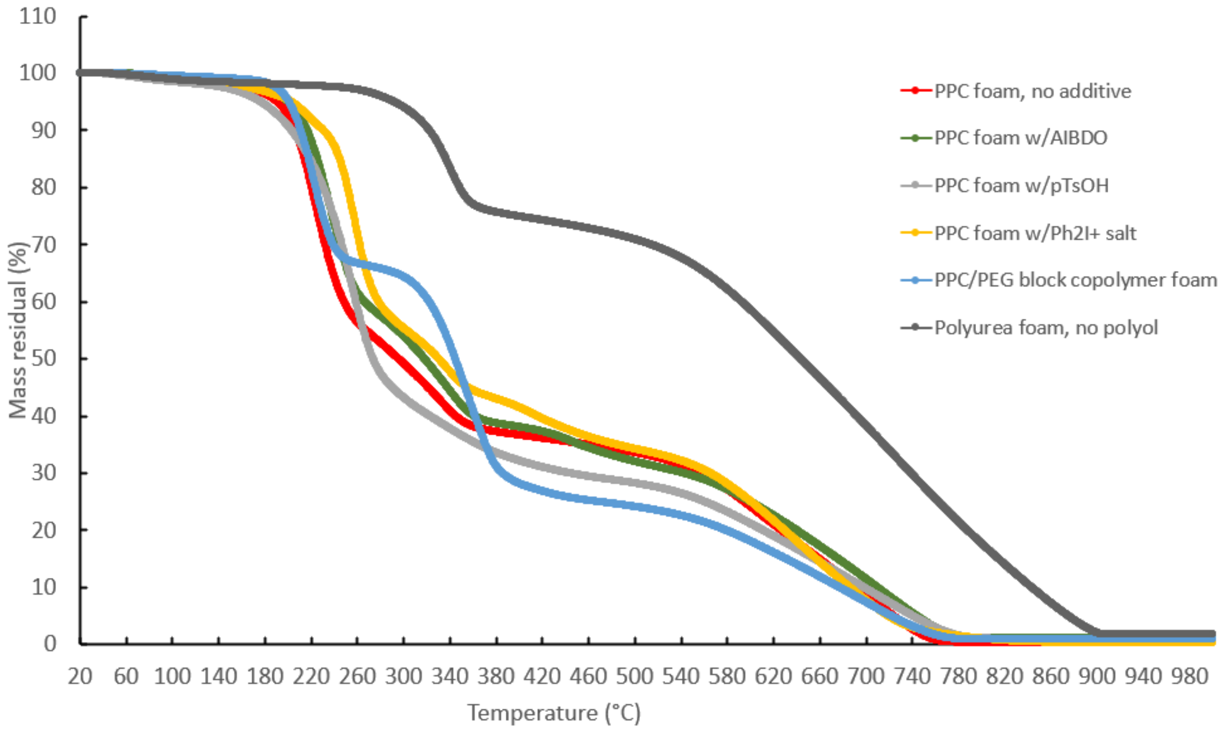

Figure S1. TGA screening of polycarbonate polyol PU foams with various additives reveals no substantial improvement in high-temperature degradation, although the strong acid pTsOH (grey) causes a lower onset of degradation. By comparison to the TGA of a foam formulated with no polyol (black), it can be seen that polyurea components dominate the high-temperature regime of degradation.

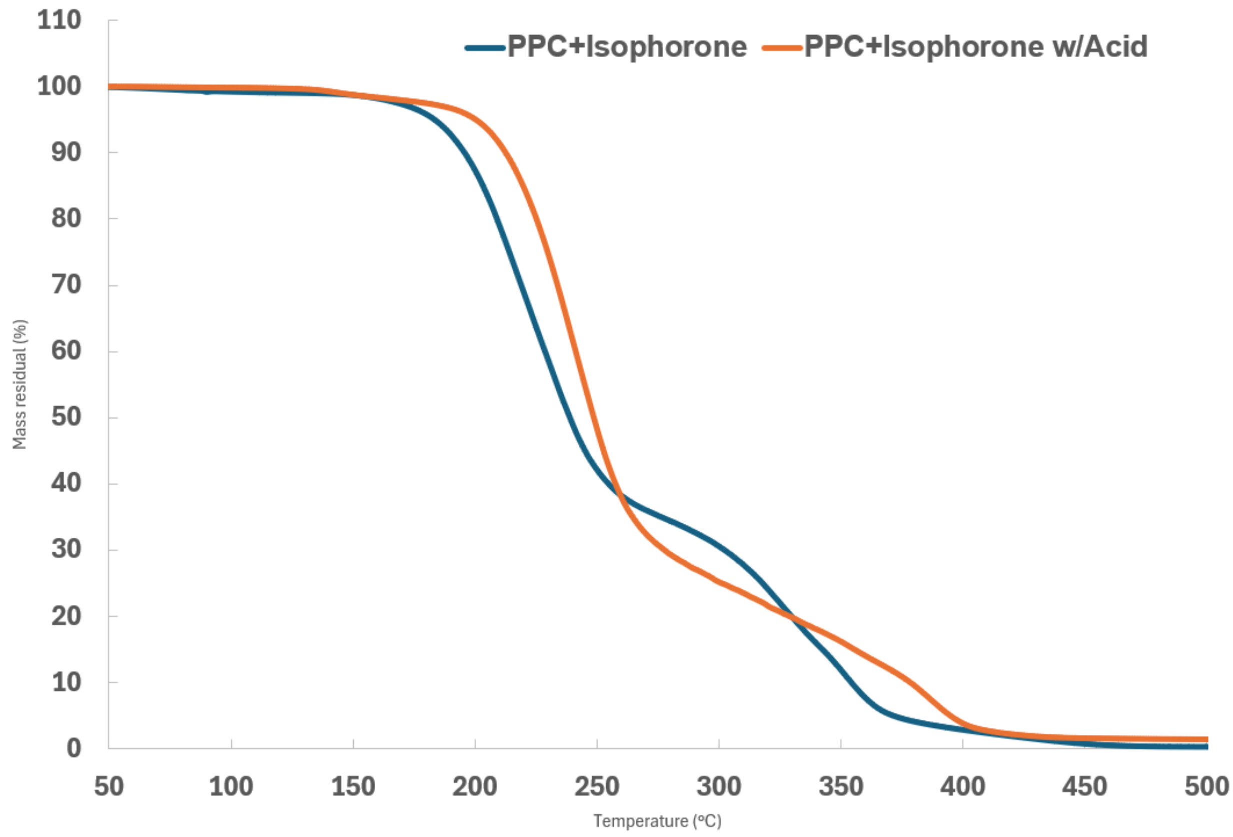

Figure S2. PPC foams with and without pTsOH formulated with the fully aliphatic diisocyanate IPDI perform similarly to foams prepared with the benzylic diisocyanate TMXDI, but with acid-free foam outperforming acid-containing foam in degradation % at most temperatures.

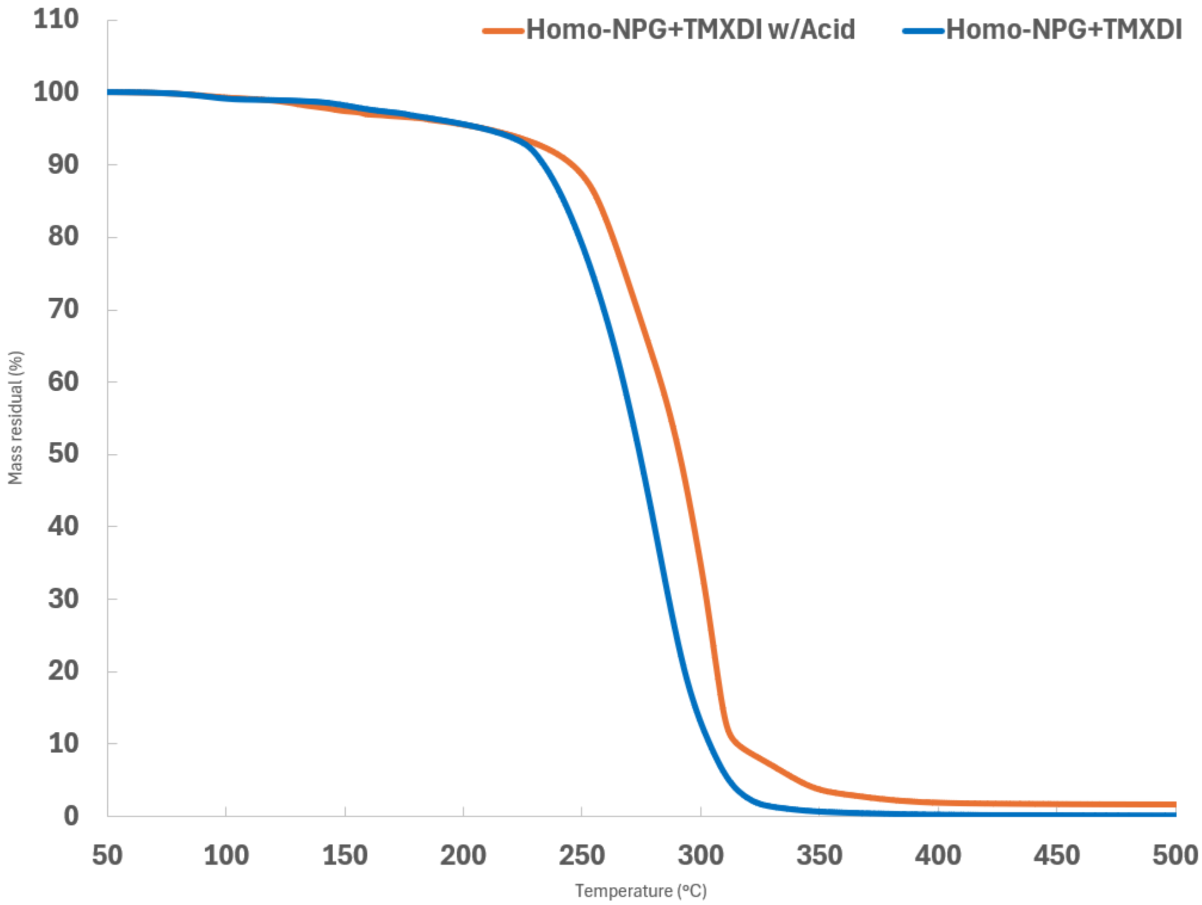

Figure S3. Foams formulated with the homopolycarbonate polyol of NPG, even with TMXDI isocyanate, degrade more readily in the absence of acid than the presence of acid at all temperatures.

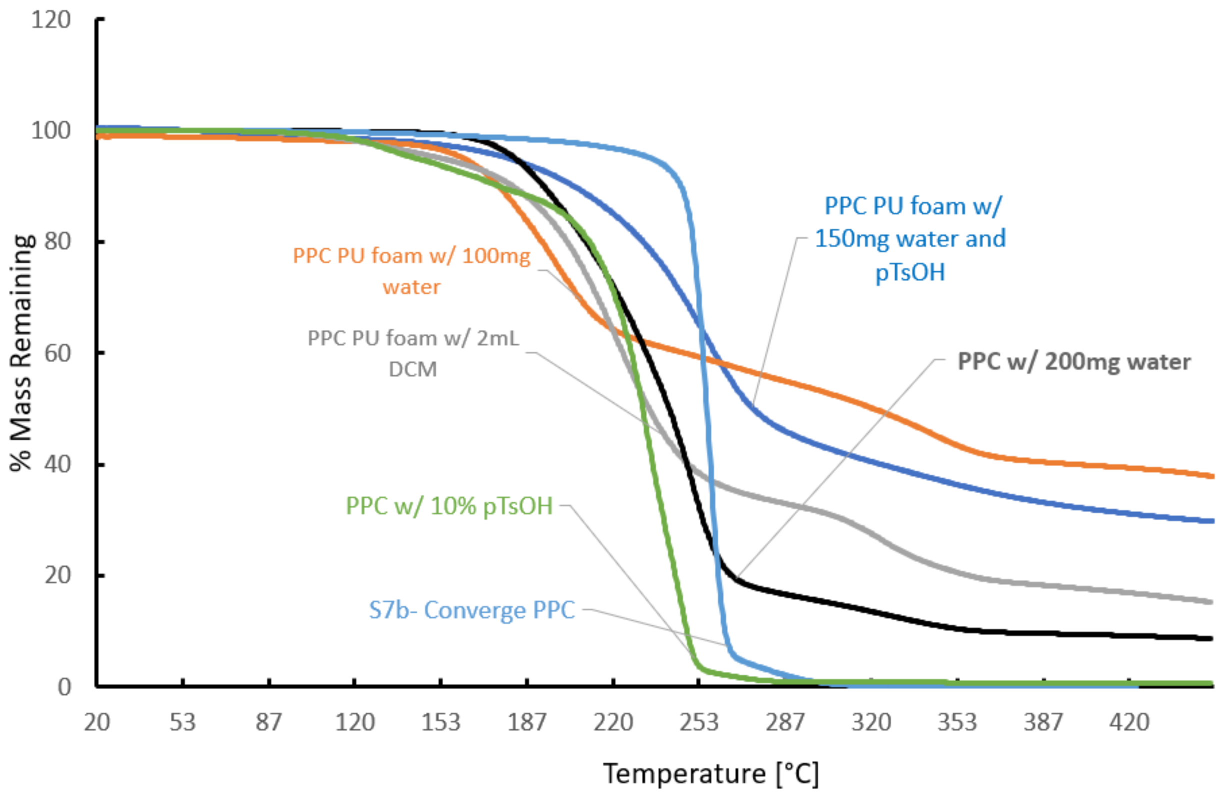

Figure S4. TGA curves for a commercial PPC polyol with and without catalytic pTsOH, and PU foams formulated with that polyol with and without DCM blowing agent.

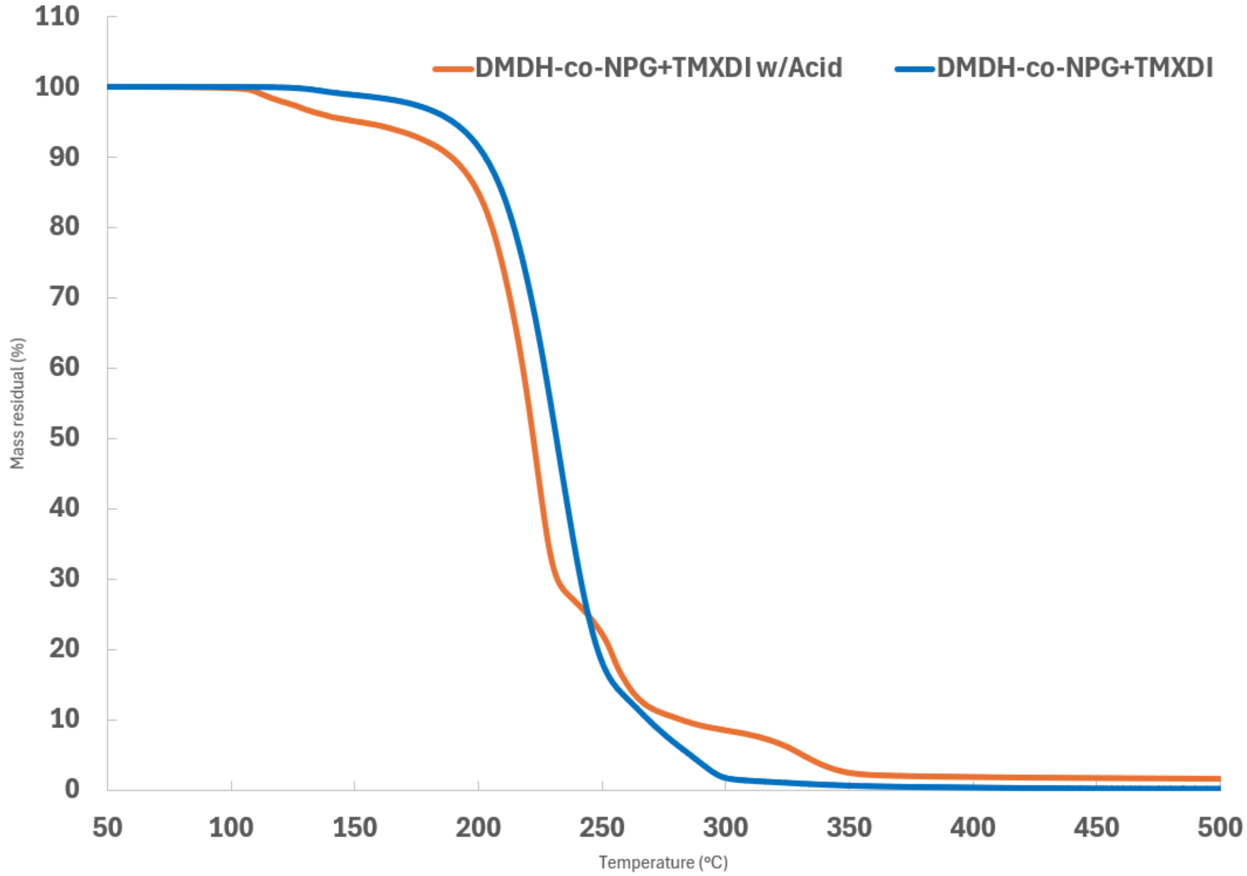

Figure S5. Foams formulated with the tertiary aliphatic polycarbonate polyol DMDH-co-NPG degrade more readily in the presence of acid than without additional acid.

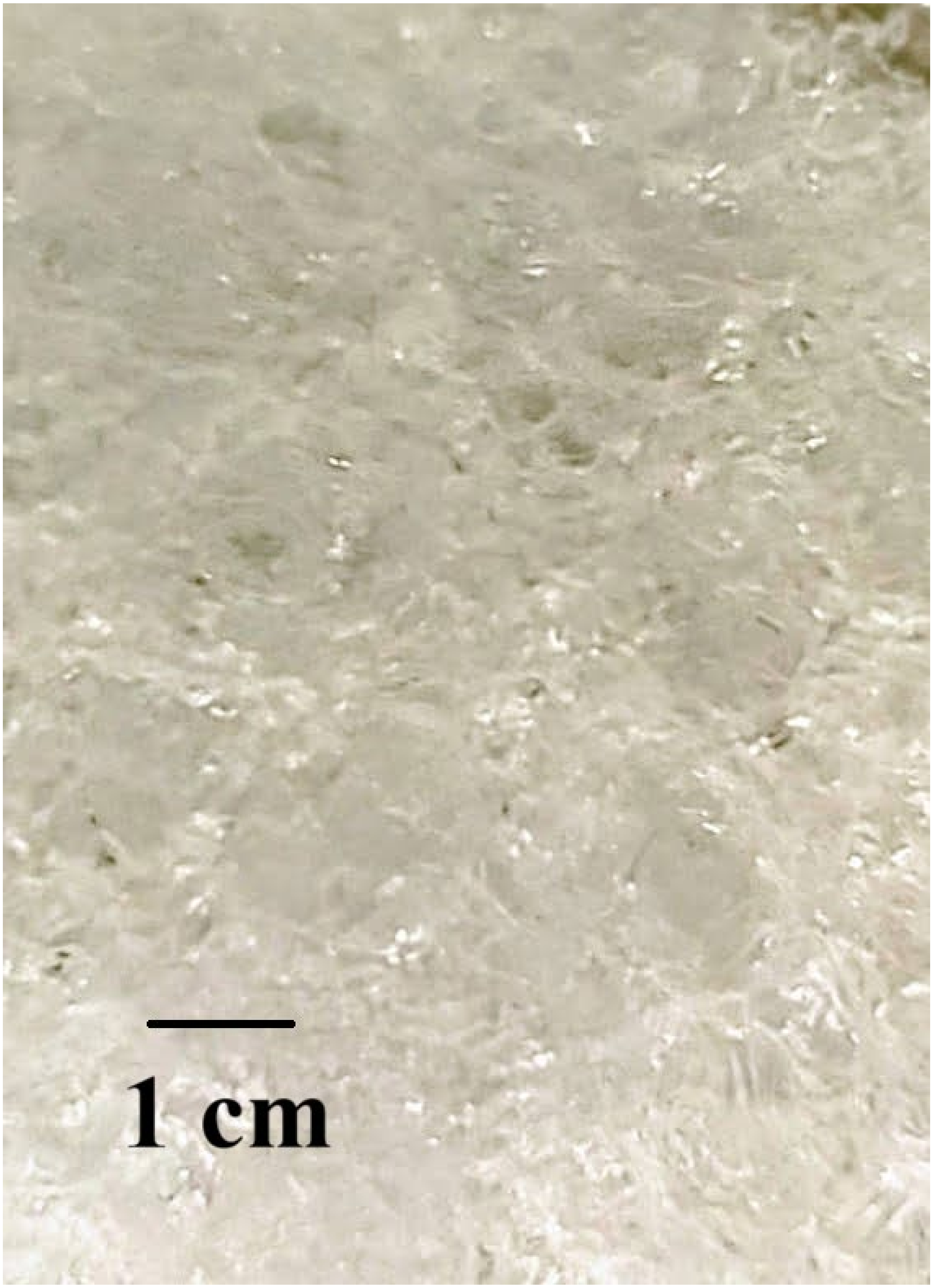

Figure S6. Macro-lens photography of a representative foam (PPC/Isophorone) shows a large, open-cell structure.

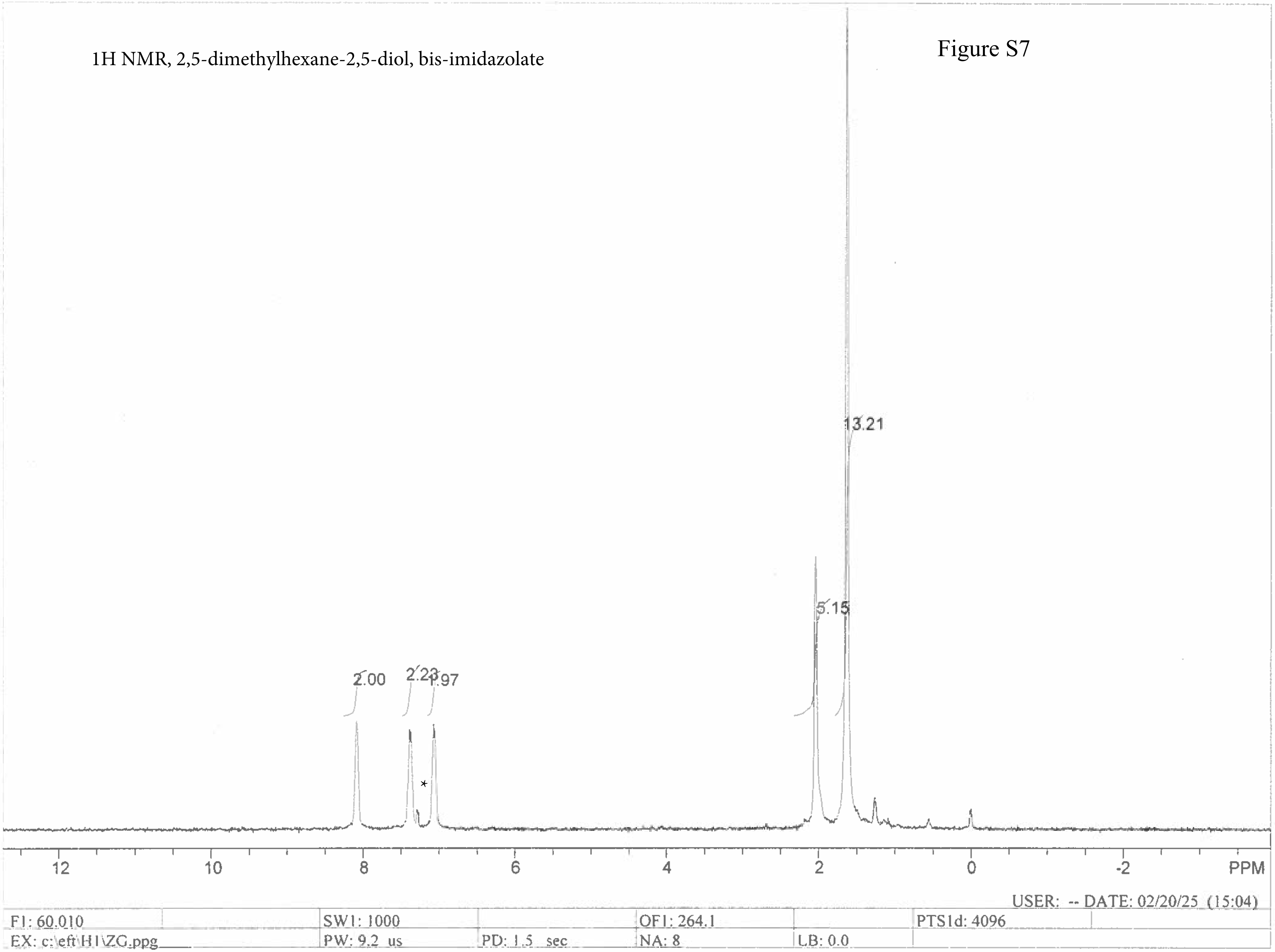
1H NMR, 2,5-dimethylhexane-2,5-diol, bis-imidazolate
Figure S7
13.21
5.15
2.00
2.23
1.97
*
12
10
8
6
4
2
0
-2
PPM
USER: -- DATE: 02/20/25 (15:04)
F1: 60.010
SW1: 1000
OF1: 264.1
PTS1d: 4096
EX: c:\eft\H1\ZG.ppg
PW: 9.2 us
PD: 1.5 sec
NA: 8
LB: 0.0

13C NMR, 2,5-dimethylhexane-2,5-diol, bis(imidazolate)

Figure S8

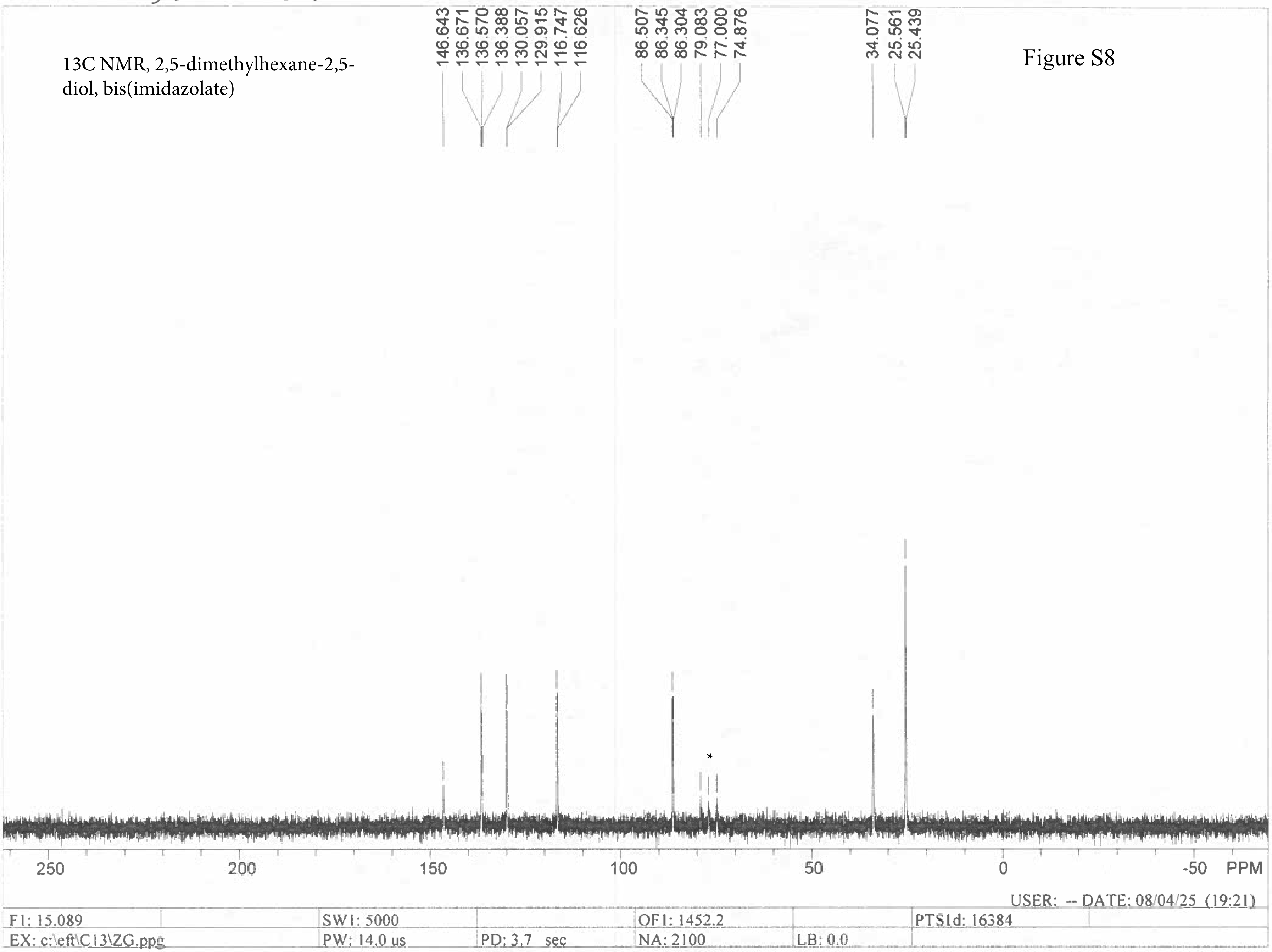

NPG imid

1H NMR, neopentyl glycol bis(imidazolate)

Figure S9

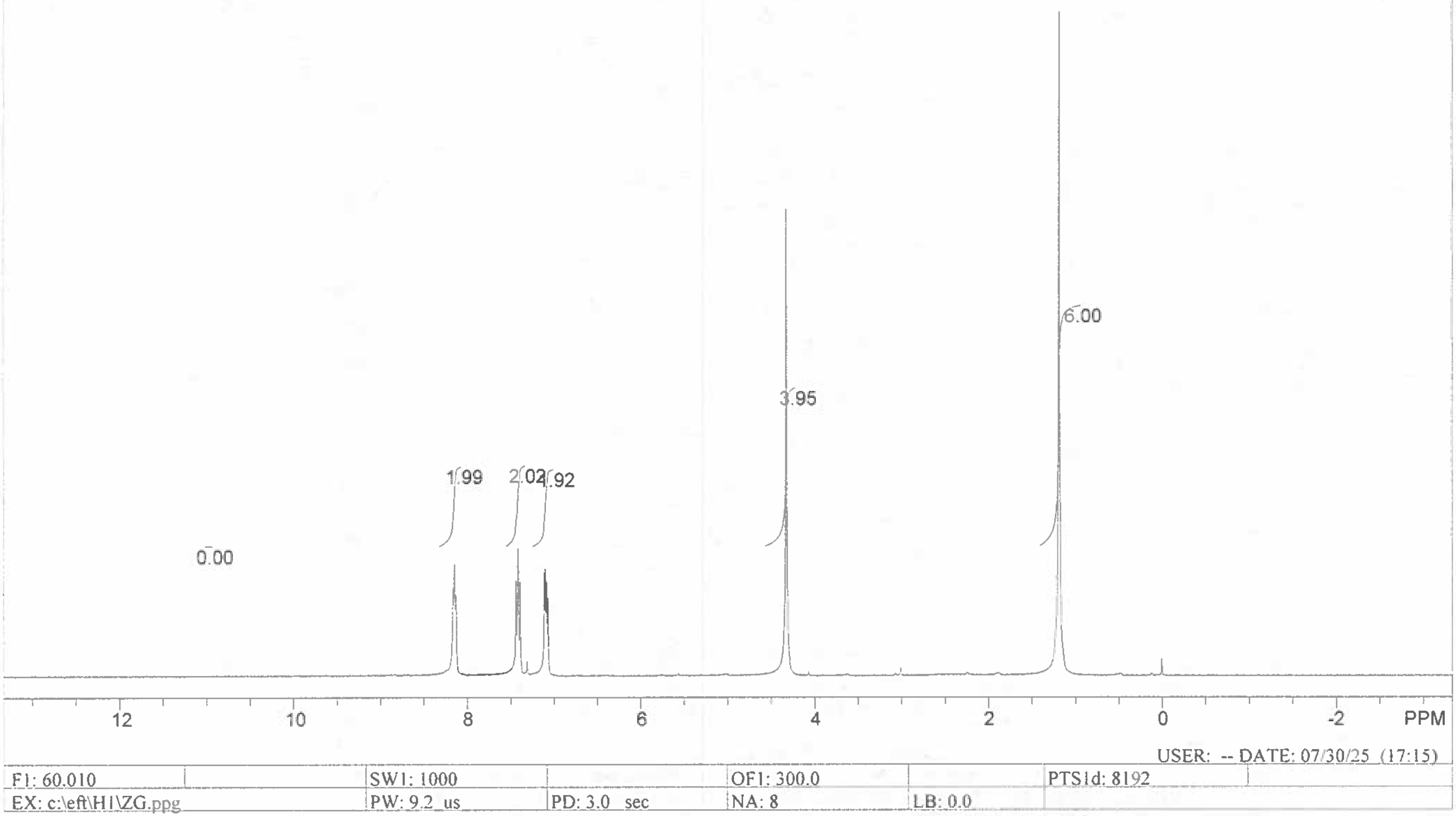

NPG imidazolate + 150 crude, 2x H2O extr

1HNMR, neopentyl glyol bis(imidazolate) crude

Figure S10

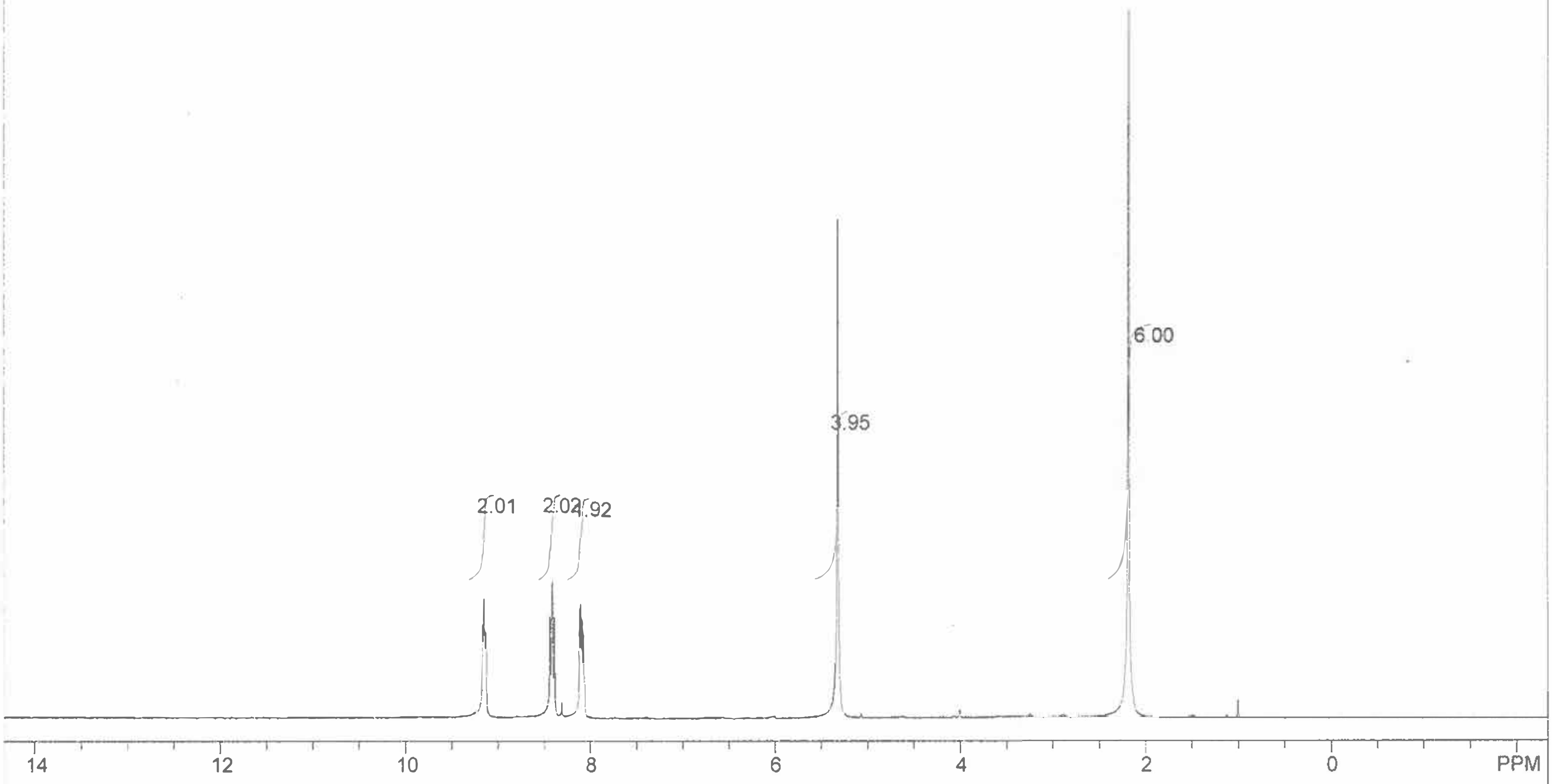

USER: -- DATE: 07/30/25 (17:15)

F1: 60.010		SW1: 1000		OF1: 360.0		PTS1d: 8192
EX: c:\eft\H1\ZG.ppg		PW: 9.2 us	PD: 3.0 sec	NA: 8	LB: 0.0	

NiG imidazolate

13C NMR, neopentyl glycol bis(imidazolate)

Figure S11

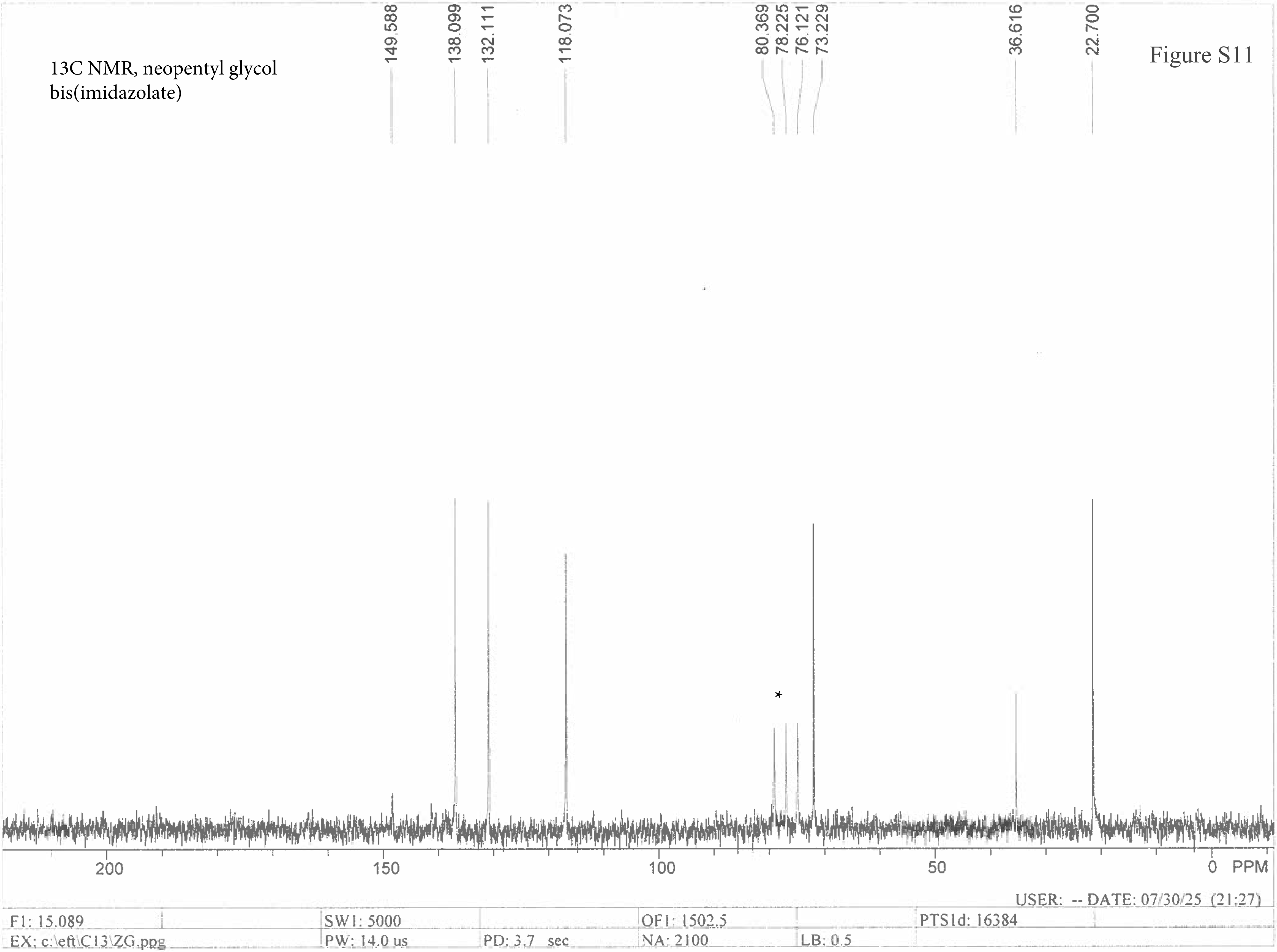

triturated diiso. benzene imidazolate

1H NMR, 1,4-benzenediisopropanol bis(imidazolate)

Figure S12

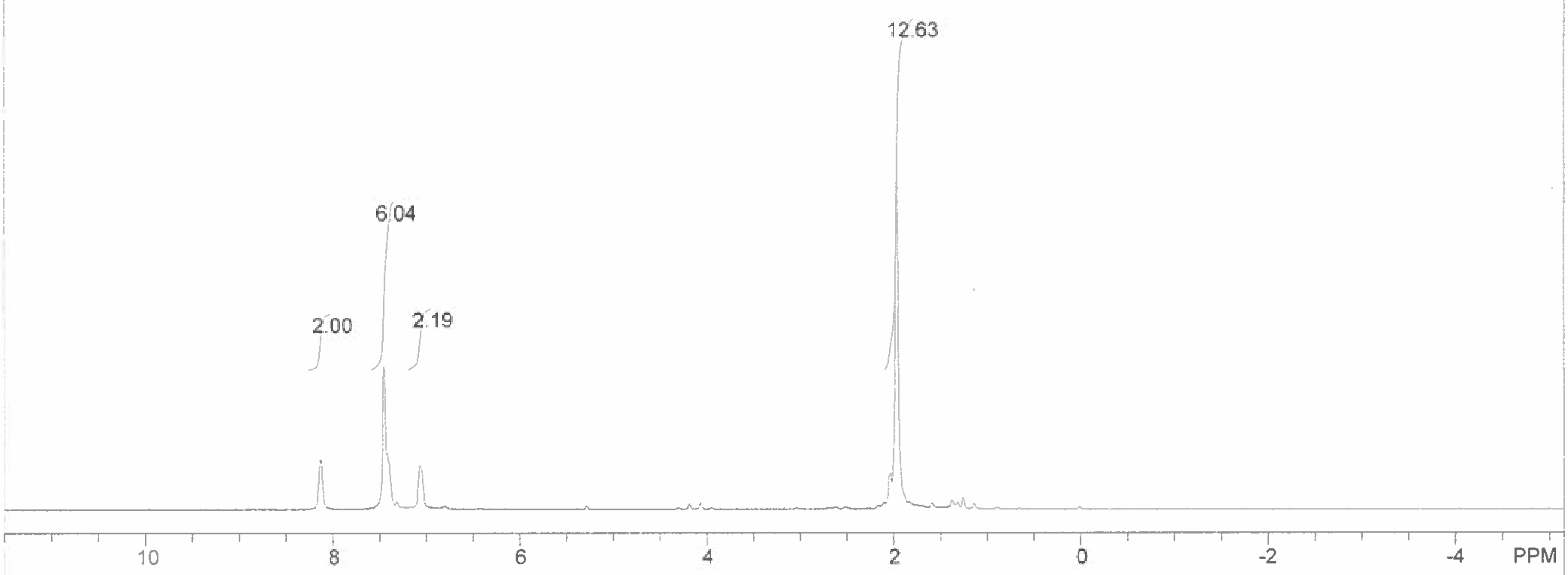

USER: -- DATE: 08/01/25 (17:39)

F1: 60.010	SW1: 1000		OF1: 190.5		PTS1d: 8192
EX: c:\eft\H1\ZG.ppg	PW: 9.2 us	PD: 3.0 sec	NA: 4	LB: 0.0	

triturated diiso. benzene imidazolate

13C NMR, 1,4-benzenediisopropanol bis(imidazolate)

Figure S13

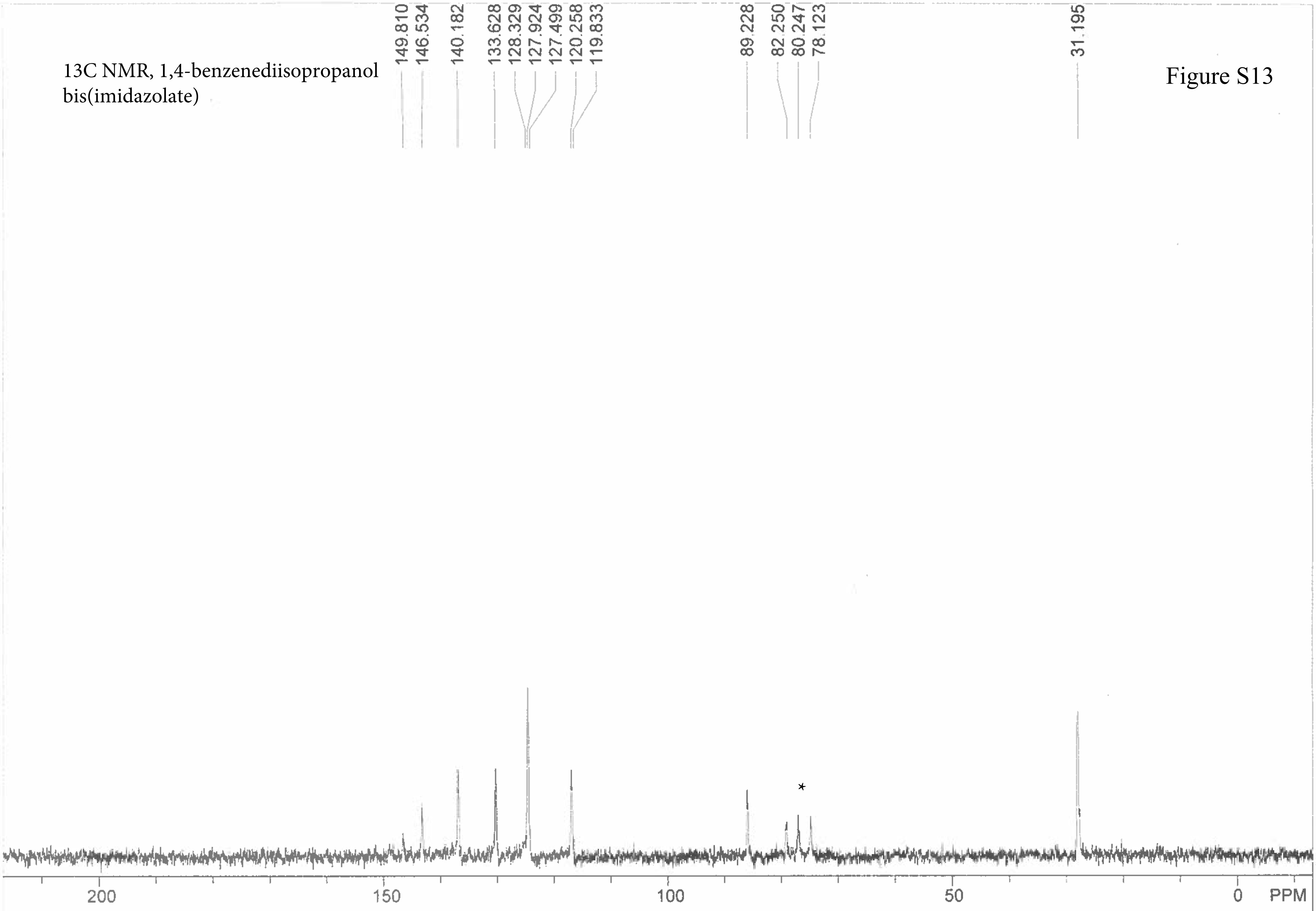

USER: -- DATE: 08/01/25 (21:52)

F1: 15.089	SW1: 5000		OF1: 1472.0		PTS1d: 16384
EX: c:\eft\C13\ZG.ppg	PW: 14.0 us	PD: 3.7 sec	NA: 2100	LB: 0.5	

Figure S14

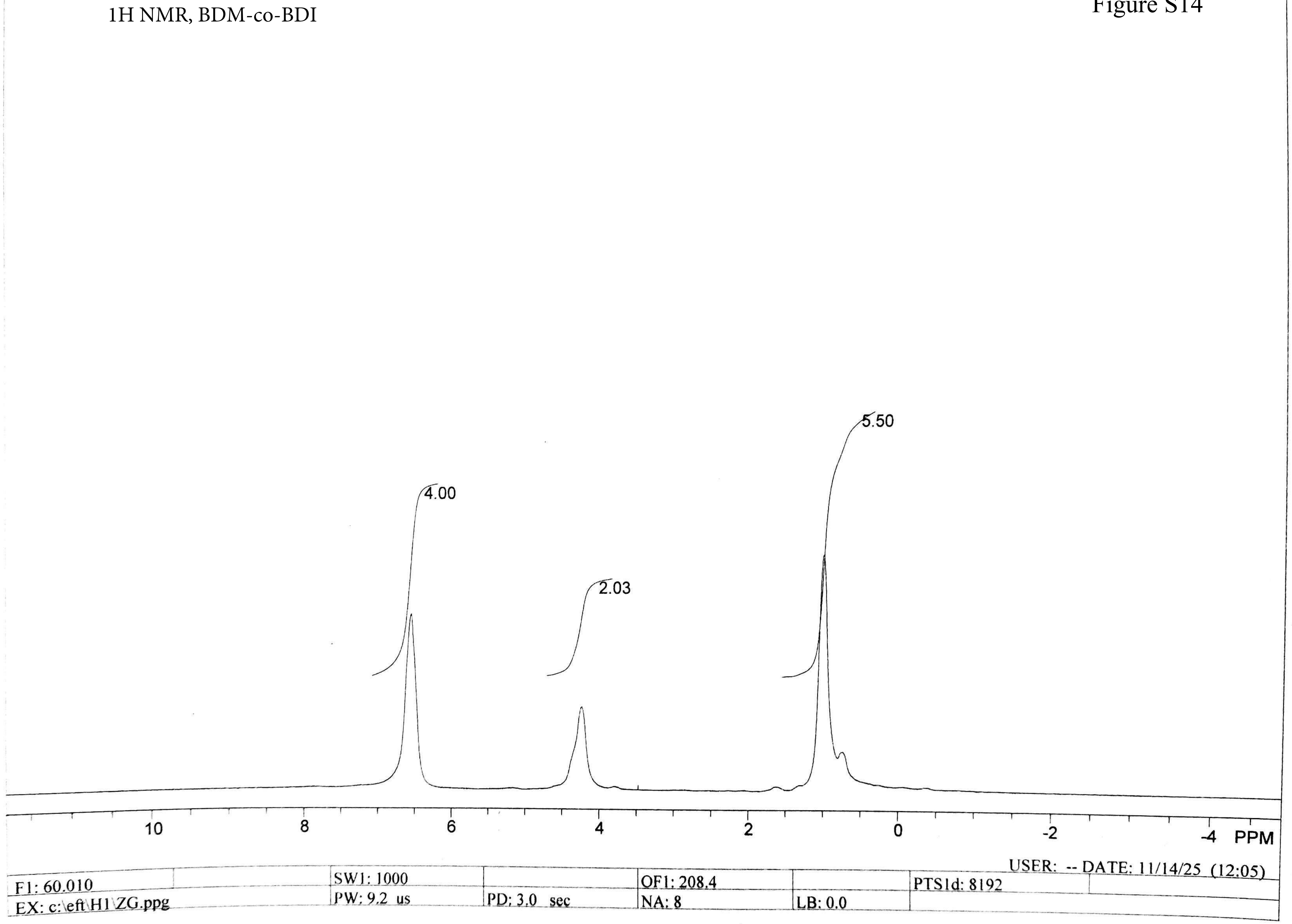
1H NMR, BDM-co-BDI
4.00
2.03
5.50
10
8
6
4
2
0
-2
-4 PPM
USER: -- DATE: 11/14/25 (12:05)
F1: 60.010
EX: c:\eft\H1\ZG.ppg
SW1: 1000
PW: 9.2 us
PD: 3.0 sec
OF1: 208.4
NA: 8
LB: 0.0
PTS1d: 8192

bdm-co-bdi

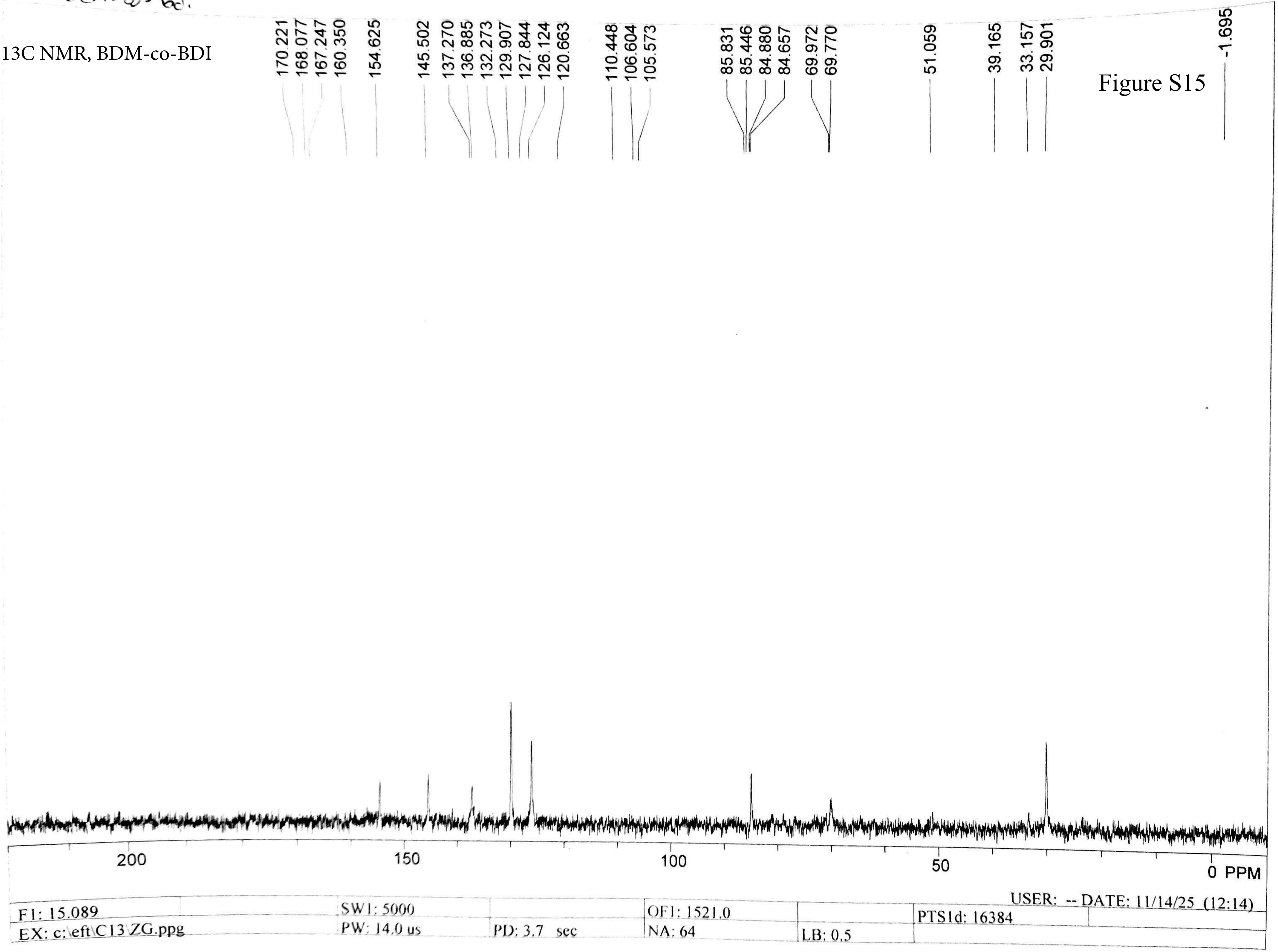

Figure S15

aliphatic polymer 1:1 CDCl3

1H NMR, NPG-co-DMDH polycarbonate polyol

Figure S16

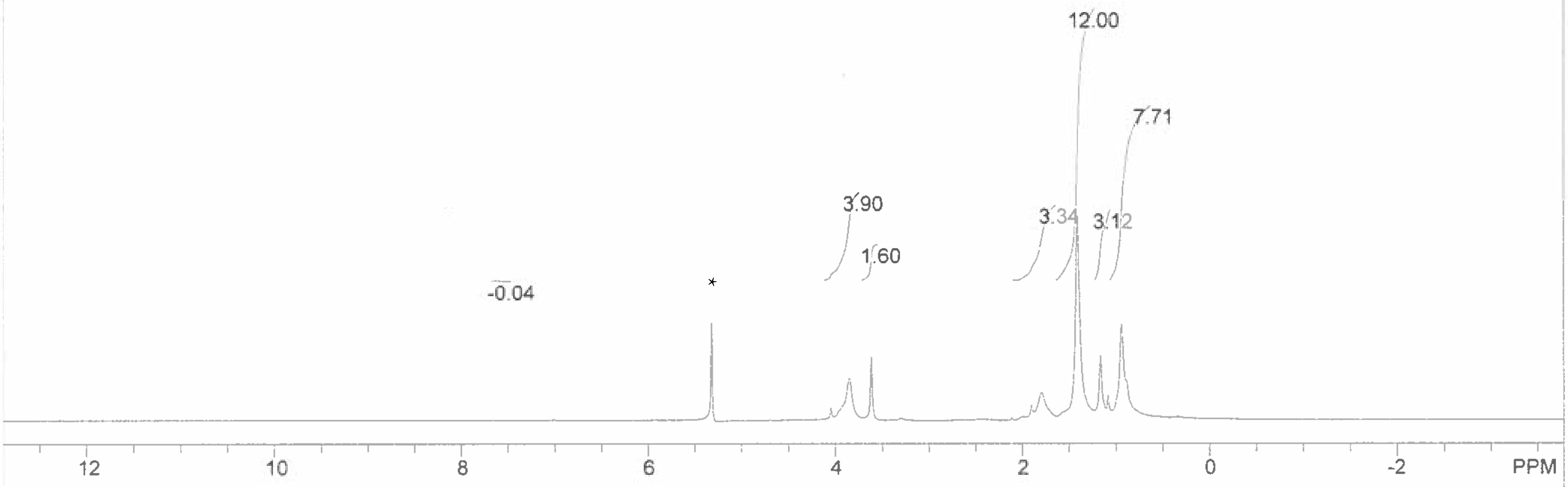

USER: -- DATE: 07/30/25 (15:08)

F1: 60.010		SW1: 1000		OF1: 273.4		PTS1d: 8192
EX: c:\eft\H1\ZG.ppg		PW: 9.2 us	PD: 3.0 sec	NA: 4	LB: 0.0	

13C NMR, NPG-co-DMDH polycarbonate polyol

Figure S17

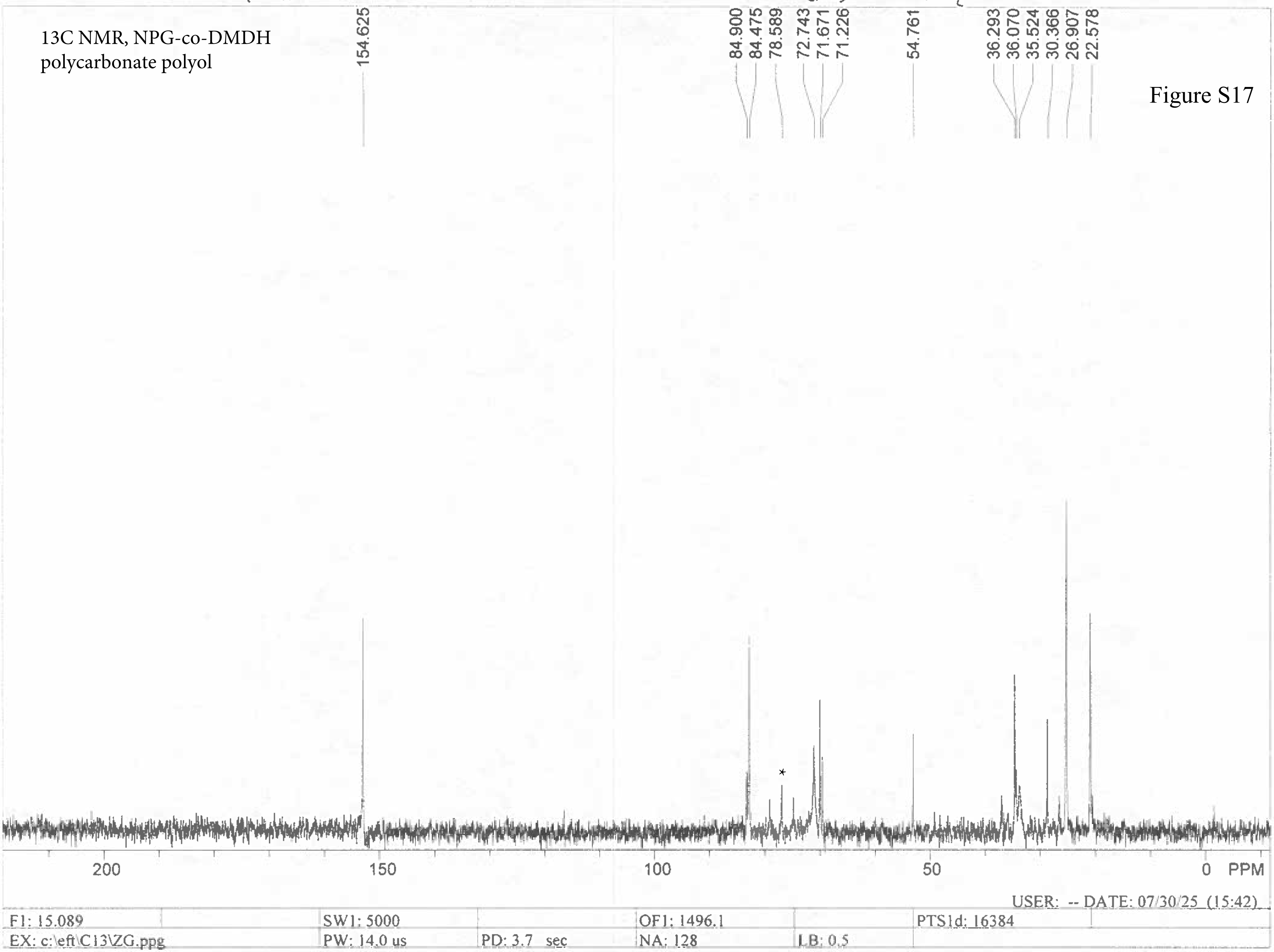

NPG homopolymer

1H NMR, NPG polycarbonate polyol

Figure S18

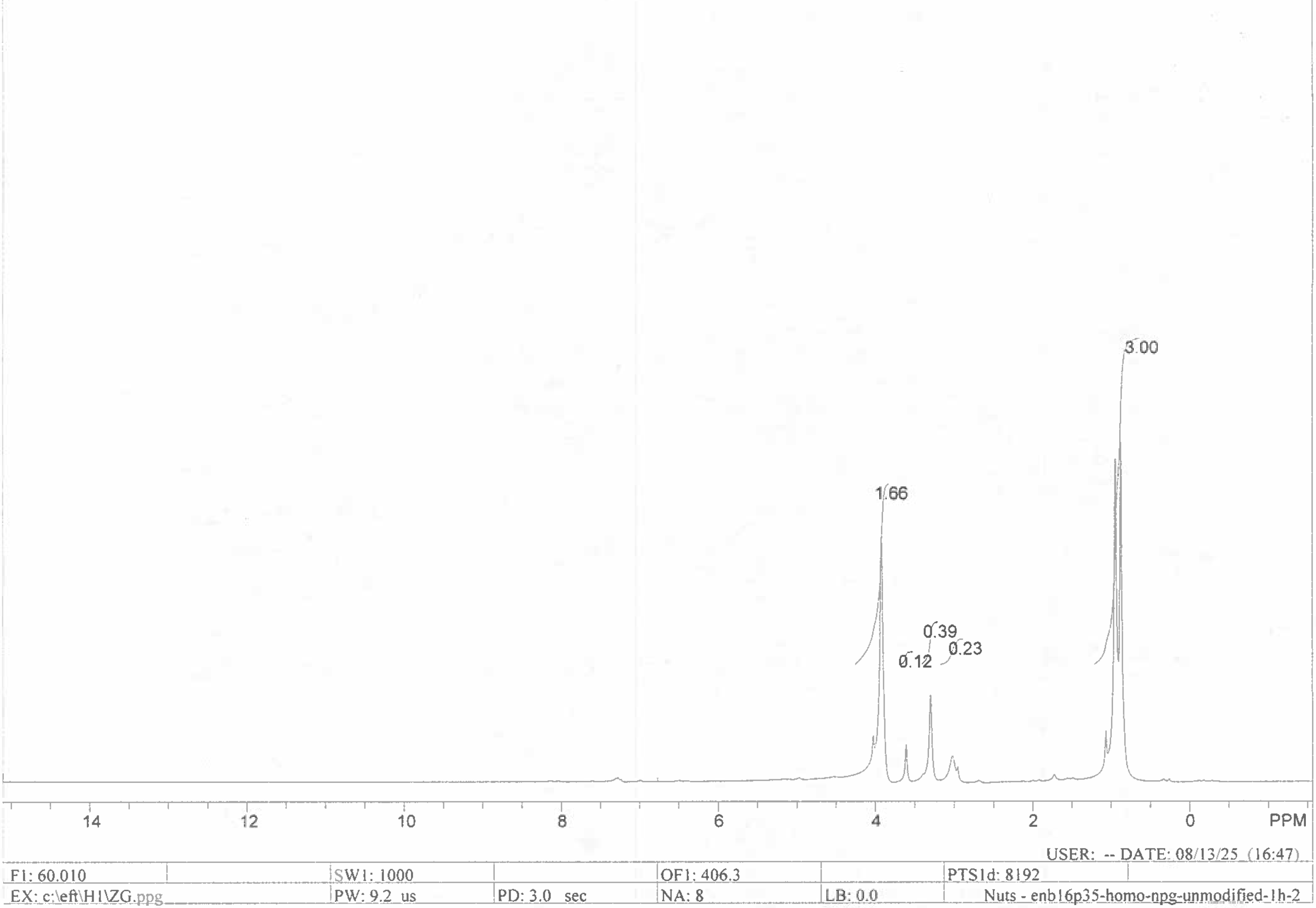

Supplement: Supplementary file 1 [file ao6c02500_si_001.pdf]
